# Unhealthy behaviors associated with mental health disorders: a systematic comparative review of diet quality, sedentary behavior, and cannabis and tobacco use

**DOI:** 10.3389/fpubh.2023.1268339

**Published:** 2024-01-05

**Authors:** Petter Grahl Johnstad

**Affiliations:** Department of Philosophy, University of Bergen, Bergen, Norway

**Keywords:** cannabis, diet quality, sedentary behavior, tobacco, mental health, mental disorder

## Abstract

**Background:**

There are well-established literatures documenting the associations between mental disorders and unhealthy behaviors such as poor diet quality, sedentary behavior, and cannabis and tobacco use. Few studies have attempted to understand the respective findings in light of each other, however.

**Objective:**

The purpose of this review was to assemble comparable data for each behavior-disorder association and assess the associations in terms of their overall strength. The review aimed to include a representative, but not exhaustive, range of studies that would allow for explorative comparisons.

**Methods:**

Eligible studies were identified via Pubmed searches and citation searching, restricted to publications no older than 2015 written in English. To obtain comparable data, only studies that reported findings as odds ratios were included, and risk of bias related to study samples, behavioral measurement disparities, and control variables was assessed via sensitivity analyses. Findings for each disorder were compared on the basis of different measures of central tendency.

**Results:**

From 3,682 records, 294 studies were included. The review found evidence of associations between each of the four unhealthy behaviors and psychosis, depression, anxiety, bipolar disorder, attention-deficit/hyperactivity disorder (ADHD), and post-traumatic stress disorder (PTSD), while personality disorder was only investigated in relation to cannabis and tobacco use. In overall comparison, the associations were generally of similar strength, and only the association between cannabis use and personality disorder was exceptional in terms of being significantly stronger than its counterparts across disorders and across behaviors. Analyses of bias risk identified some influence from behavioral measurement disparities and lack of adequate statistical control, but findings were generally robust across a range of sensitivity analyses.

**Conclusion:**

This explorative and comparative review found that poor diet quality, sedentary behavior, and cannabis and tobacco use are about equally strongly associated with a range of different mental disorders. Given the general nature of these associations, we should probably understand them to reflect a general and shared etiology. However, the findings in this review should be regarded as tentative until confirmed by more comprehensive investigations.

## Highlights

- In terms of mental disorder, moderate cannabis use appears to be about as harmful as moderate tobacco use.
- In terms of mental disorder, moderate cannabis use appears to be about as harmful as watching TV for more than 2–4 h per day or eating fast food and drinking sugary beverages every day.
- The association between cannabis use and psychosis is not stronger than the associations between cannabis use and other mental disorders.
- The association between psychosis and cannabis use is not stronger than the associations between psychosis and poor diet quality, sedentary behavior, or tobacco use.

## Introduction

Associations between unhealthy behaviors and mental disorders are well established in research literature. This review considers four different unhealthy behaviors—cannabis use, tobacco use, sedentary behavior, and poor diet quality—and a range of mental disorders across the range from common mental disorders (CMDs) to serious mental illnesses (SMIs) and including both internalizing and externalizing disorders. Aiming for a broad but not exhaustive range of disorders that can provide a convincing basis for comparison, the seven disorders psychosis, depression, anxiety, bipolar disorder, personality disorder, attention deficit/hyperactivity disorder (ADHD), and post-traumatic stress disorder (PTSD) were included in the review.

### Psychosis

Several recent reviews have confirmed an association between psychosis and cannabis use (1–6), while a few have failed to identify a statistically significant relationship (7, 8). The strength of associations identified in meta-analyses ranged from strong for the most severe cannabis users [(5): OR 3.90] to moderate [(8): OR 1.786; (3): RR 1.71; (4): OR 1.75 for abuse/dependence; (5): OR 1.97 for any use] or weak [(7): RR 1.11; (4): OR 1.14 for lifetime use]. Several recent reviews have also confirmed an association between psychosis and tobacco use (8–11), with the strength of the association ranging from moderate to strong in meta-analyses (respectively, OR 2.22, 3.04, 3.22/2.18 for case-control/prospective studies, and RR 1.99). One review did not find evidence of any significant association (12). Those reviews that have compared the strength of the relationship between cannabis and psychosis to that between tobacco and psychosis have found that the latter appears to be somewhat stronger (8, 13). The associations between psychosis and poor diet quality or sedentary behavior have not been extensively studied, although reviews have suggested that people with psychosis tend to have poor diet quality (14, 15) and low levels of physical activity (16, 17).

### Depression

A number of recent review articles support an association between cannabis use and depression, although with some mixed results (12, 18–28), while one found that the relationship is unclear (29). The meta-analysis by Onaemo et al. (26) identified a strong association between cannabis use disorder and major depression (OR 3.22), while those by Esmaeelzadeh et al. (18) and Gobbi et al. (19) as well as an earlier meta-analysis by Lev-Ran et al. (30) identified a weak to moderate association (respectively, OR 1.29, 1.37 and 1.62) for overall cannabis use.

For tobacco, recent reviews by Weinberger et al. (31) and Fluharty et al. (32) confirmed that people with depression are more likely to smoke and to meet criteria for nicotine dependence, with the latter also indicating that smoking may lead to depression. A meta-analysis by Esmaeelzadeh et al. (18) found that the association between tobacco use and depression was moderately strong with an OR of 1.65, which was stronger than that for the corresponding association between cannabis use and depression. Another meta-analysis by Groenman et al. (33) found that childhood depression was a risk factor for subsequent nicotine use disorder (OR 2.56). Reviews by Zeng and Li (34) and Han et al. (35) also confirmed that secondhand smoking is associated with depression, with their meta-analyses obtaining ORs of 1.60 and 1.32, respectively. Furthermore, recent reviews have also indicated that e-cigarette use is associated with depression (36, 37).

There are also a number of reviews supporting associations between depression and sedentary behavior (17, 38–47) and poor diet quality (48–57), although with some mixed results. In meta-analyses, the associations were weak to moderate in strength, with RR 1.42 (47) and RR 1.25 (46) for overall sedentary behavior, OR 1.28 (45) and OR 1.12 (42) for screen time, RR 1.18 for TV viewing (41), OR 1.44 for ultra-processed food consumption (49), RR 1.25 (58) and RR 1.31 (48) for sugar-sweetened beverage consumption, OR 1.18 for western-style unhealthy dietary pattern (59), and OR 1.62 (52) and OR 1.11 (54) for fast/junk food.

### Anxiety

Some reviews have identified an association between cannabis use and anxiety (18, 20, 26, 27, 60–62), although others have maintained that the relationship is unclear (19, 22, 28, 29, 63, 64). In meta-analyses, moderate to strong associations were identified between cannabis use disorder and anxiety [(60): OR 1.68; (26): OR 2.99], while the associations for overall use were generally weak [(18): OR 1.36; (19): OR 1.18; (60): OR 1.24; (61): OR 1.15; (62): OR 1.25]. Reviews have also identified an association between tobacco use and anxiety (12, 18, 65–67), although with some mixed results. A meta-analysis by Esmaeelzadeh et al. (18) found a strong association between tobacco use and anxiety with an OR of 2.21, which was stronger than that for the corresponding association between cannabis use and anxiety.

Reviews have also found evidence of an association between anxiety and sedentary behavior (68–71) and poor diet quality (49, 52). Associations in meta-analyses were of weak to moderate strength, with regular physical activity reducing the odds for anxiety [(72): OR 0.54; (70): OR 0.74] and sedentary behavior [(68): OR 1.48], ultra-processed food [(49): OR 1.48] and junk food [(52): OR 1.24] increasing the odds for anxiety.

### Bipolar disorder

The relationship between cannabis use and bipolar disorder has not been intensively studied, but a number of reviews have suggested a positive association (20, 23, 25, 73–78). Meta-analyses by Gibbs et al. [(73): OR 2.97] and Hunt et al. [(74): OR 2.35] identified a strong association between cannabis use disorder and bipolar disorder. Some reviews have also suggested an association between tobacco use and bipolar disorder (75, 79), although the evidence is mixed (12). Nevertheless, the meta-analysis by Jackson et al. (79) identified a strong association with an OR of 3.5. Reviews have also identified tentative associations between bipolar disorder and sedentary behavior (17, 80, 81) and diet quality (82, 83), but there are few available studies.

### Personality disorders

While the association between personality disorders and general substance use is well established, the evidence specifically for cannabis (20, 28, 84–86) and tobacco (12, 87) is quite limited. Similarly, the research into the relationships between personality disorders and sedentary behavior and diet quality is very limited (87, 88).

### Attention-deficit/hyperactivity disorder

ADHD is commonly diagnosed in childhood, and most studies investigating the relationship between the disorder and substance use focus on ADHD as a risk factor for subsequent substance use. For cannabis, meta-analyses have found strong associations between childhood ADHD and regular [(89): OR 2.45] and lifetime use [(90): OR 2.78] as well as a moderate association for abuse or dependence [(90): OR 1.58]. For tobacco, meta-analyses similarly identified childhood ADHD as a strong risk factor both for subsequent nicotine use [(89) OR 2.16; (90): OR 2.08] and nicotine dependence or use disorder [(33): OR 2.52; (90): OR 2.82]. The evidence for e-cigarette use is very limited (36).

Reviews have also supported associations between ADHD and sedentary behavior (91–95) and diet quality (96–99). Meta-analyses of sedentary behavior found moderate to strong associations between electronic media use and ADHD [(91): OR 1.94; (92): 2.597], while meta-analyses of diet quality found weak to strong associations for sugar and soft drink consumption [(97): pooled effect size 1.22], junk food [(99): OR 1.51], and general unhealthy diets [(96): OR 1.41; (98): OR 2.24].

### Post-traumatic stress disorder

Reviews have identified an association between cannabis use and PTSD (100, 101) and investigated the potential for therapeutic use (102–104). Reviews have also identified an association between tobacco use and PTSD (105–109), with one meta-analysis by van den Berk-Clark et al. (109) finding a strong association (OR 2.13) for unadjusted studies and a weak association (OR 1.22) for a single adjusted study. The evidence for e-cigarette use is very limited (36).

Furthermore, reviews have suggested that both sedentary behavior and poor diet quality are associated with PTSD, although with some mixed results (70, 109–111). In meta-analyses, high physical activity was moderately protective against PTSD [(70): OR 0.57], while PTSD in turn was a weak risk factor for low physical activity [(109): OR 0.91 for adjusted studies and OR 0.69 for unadjusted studies]. The meta-analysis by van den Berk-Clark et al. (109) on diet quality was somewhat unclear, as four unadjusted studies predicted a healthier diet (OR 1.25) while a single adjusted study predicted a less healthy diet (OR 0.95).

While these reviews indicate a broad range of associations between unhealthy behaviors and mental disorders, they also demonstrate that such associations have not often been understood in relation to each other across various behaviors and disorders, which would allow for both a calibration of their relative importance and a widened perspective on their etiology. Instead, individual associations are usually analyzed and discussed in isolation, which encourages the perspective that the association under scrutiny is unique and reflects a particular and individual etiology. Should it be true that the range of disorders here under scrutiny are generally associated with a range of unhealthy behaviors related to diet, exercise, and licit and illicit substance use, however, it might seem unlikely that these parallel associations are best understood in isolation from one another. If the association between a given behavior and a given mental disorder has parallels across a range of disorders and behaviors, its lack of specificity indicates that the association probably does not reflect a causal effect from the behavior itself (112). Instead, such a field of associations most likely point to general and shared dynamics. One such dynamic may involve underlying issues of social misery and psychological trauma that perhaps give rise to both unhealthy behaviors and to mental disorders. To take one example, long-term unemployment may entail poverty, boredom, and social isolation, which in turn may push a person into unhealthy behaviors as means of escapism and diversion and also contribute to a mounting sense of hopelessness and depression.

Indeed, there is strong evidence supporting a relationship between mental disorder and factors relating to misery and trauma. To take a few noteworthy examples, childhood abuse has been associated with psychosis at ORs of 7.3 (113) and 5.9 (8), with depression at ORs of 19.1 (114) and 4.2 (115), and with ADHD at ORs of 7.3 (91) and 3.3 (116), as well as with cannabis abuse at ORs of 5.8 (117), 2.7 (118), and 2.2 (119). Violence victimization has similarly been associated with psychosis at ORs of 5.9 (120), 4.0 (121), 2.2 (122), and 1.9 (123), with PTSD and depression at ORs of 32.4 and 5.4 (124), and with nicotine dependence at ORs of 22.4 (124) and 5.5 (125). Afifi et al. (118) for their part found that living in an unsafe community was associated with cannabis use at an OR of 5.0 and with tobacco use at an OR of 3.8. Comparable findings exist for childhood bullying victimization (120, 126), while poverty, low education, and unemployment have been associated with psychosis at ORs of 8.2 (127) and 3.0 (122), with depression at ORs of 9.1 (128), 5.0 (129), 3.4 (130), 3.2 (131), 2.8 (132), and 2.6 (133), with nicotine dependence at ORs of 37.0 (134), 2.4 (135), and 2.3 (125), and with cannabis use disorder at an OR of 2.5 (136). It should also be noted that factors relating to trauma and misery might accumulate in an individual and thereby increase risk. In a study by McMahon et al. (120), cumulative adverse life events were strongly associated with psychotic experiences, with four or more adverse life events incurring an OR of 16.8.

Thus, it seems possible that both unhealthy behaviors and mental disorders might be caused by underlying problems related to social misery and psychological trauma, and that the observed associations between those behaviors and disorders are largely spurious. As a basis for the discussion of the specificity of individual associations, this article will use the probably most discussed associations among those here under purview, namely that between cannabis use and psychosis, which is commonly taken to reflect a causal effect from cannabis use. A recent consensus paper by D'Souza et al. (1) pointed to a study by Starzer et al. (137) as evidence supporting the specificity of this association, and on this basis drew the inference that “[c]annabis is more likely to be associated with psychosis outcomes than other psychiatric diagnoses” (p. 732). This consensus paper also pointed to a somewhat broader range of evidence to support the specificity of the cannabis-psychosis association as compared to other forms of substance use, drawing the inference that “[t]he risk for a psychosis outcome is highest for cannabis relative to other drugs” (ibid.).

However, there is also evidence contradicting the consensus view presented in D'Souza et al. (1). As Ksir and Hart (138) pointed out, research has associated cannabis use with depression, which may challenge the specificity of the cannabis-psychosis association. Furthermore, a recent review by Johnstad (13) found that tobacco use tended to be at least as strongly associated with psychosis as is cannabis use. The cannabis-psychosis association may therefore lack specificity both across disorders and across behaviors, which would open for the perspective that the association may be spurious and reflective of a general etiology perhaps related to underlying psychological trauma and social misery.

This interpretation is supported by a study by Shakoor et al. (139) which found that in a sample of adolescent twins, “[e]nvironmental influences explained all of the covariation between cannabis use and paranoia, cognitive disorganization and parent-rated negative symptoms,” with a bivariate common environment of 69%−100% (p. 144). Cannabis use explained 2%−5% of variance in positive, cognitive, and negative psychotic experiences. Sideli et al. (140) for their part found that less than daily cannabis use was not associated with psychosis (OR 0.97, *p* = 0.92) unless it co-occurred with having experienced child abuse, in which case the association was strong (OR 2.51, *p* < 0.05), while Copeland et al. (141) found that cannabis use before the age of 16 was strongly associated with depression (OR 2.3) and anxiety (OR 2.9) in an analysis adjusted only for sex and race/ethnicity, but that the association disappeared with adjustment for childhood disorders and adversities (OR 0.6 for both depression and anxiety). A meta-analysis by Fusar-Poli et al. (8) similarly found that risk for psychosis was related to environmental risk factors such as childhood trauma and further accumulated by low education and unemployment, while finding no significant impact from cannabis use.

In sum, the worry that unhealthy behaviors such as cannabis use cause psychosis may be a case of projecting the social characteristics of the drug-using population onto the drug itself and understand these characteristics as pharmacological effects inherent to the drug use. This comparative review aimed to assemble data on a broad range of associations between unhealthy behaviors and mental disorders in order to compare their respective strengths and to gain insight into the influence of factors relating to trauma and misery by recording the inclusion of relevant control variables and subsequently running sensitivity tests on the dataset. It reviews recent (2015 through January 2023) research on associations between a range of unhealthy behaviors and mental disorders, using the much-debated association between cannabis use and psychosis as a basis for comparison. In order to obtain comparable figures, the review included only records that presented their findings as odds ratios, and attempted to account for variations in behavior intensity, sample generalizability, and covariates via subsequent sensitivity analyses. By thus gaining a comparative overview of the relationship between unhealthy behaviors and mental disorders, the review hoped to achieve three specific aims:

1. Understand individual associations between a given unhealthy behavior and a given mental disorder in relation to other such disorders.
2. Understand individual associations between a given unhealthy behavior and a given mental disorder in relation to other such behaviors.
3. Understand the association between unhealthy behaviors and mental disorders in relation to statistical control for covariates.

## Methods

The pre-planned criteria for inclusion were that studies must investigate associations between any of the relevant behaviors and disorders and report their findings as odds ratios in a manner that allows for cross-study comparisons. In order to keep the number of included studies manageable, only recent studies (no older than 2015) would be included. Furthermore, the studies should be published in English, be available in the Pubmed database, and their sample size should be at least 100.

Two Pubmed searches were performed (February 3, 2023). The search related to cannabis and tobacco use was [(tobacco OR nicotine OR cigarette OR cannabis OR marijuana) AND (psychosis OR depression OR anxiety OR bipolar OR “personality disorder” OR adhd OR ptsd) AND associat^*^ AND (use OR “use disorder” OR dependence OR abuse) AND (comorbid OR dual OR psychiatric)] while the search related to diet quality and sedentary behavior was [(“poor diet” OR “sedentary behavior” OR “fast food” OR soda OR “sugar-sweetened”) AND (psychosis OR depression OR anxiety OR bipolar OR “personality disorder” OR adhd OR ptsd) AND associat^*^]. The author assessed all records. Reported associations in included studies were classified as cross-sectional or longitudinal, with retrospective analyses being classified as longitudinal. In order to investigate directionality as openly as possible, longitudinal studies of both unhealthy behaviors leading to mental disorders and mental disorders leading to unhealthy behaviors were included. Study quality was assessed on the level of sample representativeness, behavioral measurement disparities, and the inclusion of control variables related to social misery and psychological trauma. Results are reported according to PRISMA guidelines (142).

**Figure 1:**
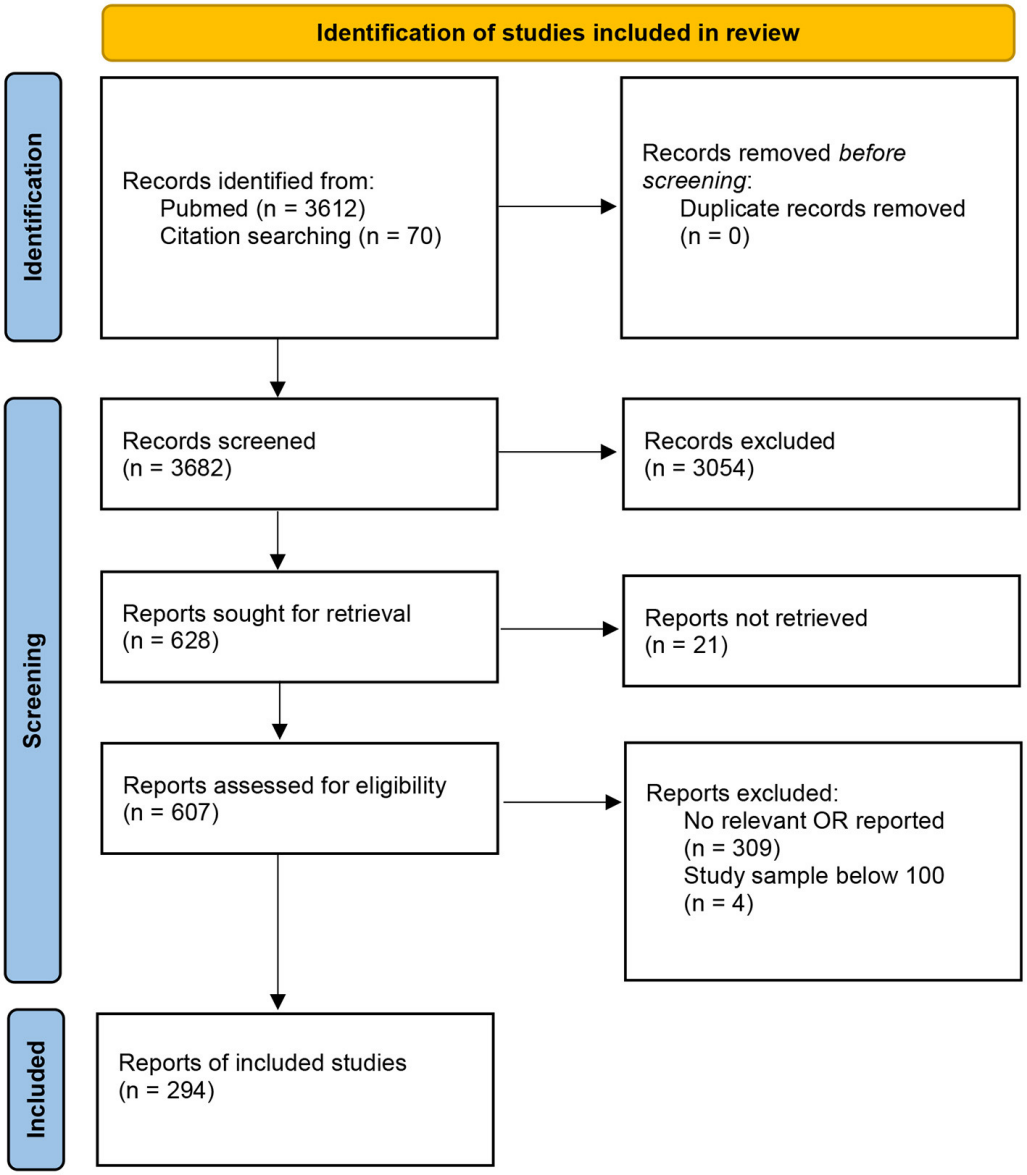
Flow diagram for article inclusion.

The selection procedure is shown in the flow chart of Figure 1. Criteria related to language and publication date were implemented in the search itself. Four reports were excluded for having samples lower than *n* = 100, while 309 reports were excluded because they did not report any relevant odds ratios that allowed for comparisons. This was related to the following reasons:

1. The reports combined several types of drugs or disorders into a single construct (e.g., “substance use,” “psychological distress,” “internalizing disorders”).
2. The reports subdivided disorders into specific symptoms or symptom classes (e.g., auditory and visual hallucinations in psychosis).
3. Findings were reported in other terms than odds ratios (e.g., risk ratios, hazard ratios).
4. Some reports especially of sedentary behavior were also excluded because they reported ORs for continuous variables that were not readily comparable to the other studies included in the review.
5. A few reports utilizing Mendelian randomization methodology were excluded because they were not comparable to the cross-sectional or longitudinal studies included in the review and did not constitute a meaningful basis for comparison on their own.

### Variables

The review focused on moderate cannabis and tobacco use as compared to high levels of sedentary behavior and poor diet quality. This approach was founded on the observation in Johnstad (13) that heavy substance use is very likely associated with underlying factors of social misery and psychological trauma, and that analyses of the heavy drug-using population are therefore at high risk of confusing the social characteristics of this population segment with the effects from the drug use itself. While heavy chronic drug use is commonly driven by coping motives related to underlying misery and trauma, moderate use is driven by a much broader range of motivations (143–145). In order to understand pharmacological effects from substance use in and of themselves, it therefore seems advisable to focus on moderate usage patterns.

In this review, moderate cannabis use was defined as one or two use occasions per week (146, 147). For tobacco, moderate use could be defined as daily use of about 10 cigarettes (148–150). When the studies included in this review reported effects related to several different usage patterns, the one most closely resembling a moderate pattern was selected. The review did not exclude any study on the basis of divergences in the usage pattern, however, and several included studies focused exclusively on use disorders or similarly problematic behavior. For sedentary behavior and diet quality, the review focused on high exposure represented by such measures as the worst quartile, daily consumption of soda or fast-food, not meeting physical activity guidelines, or more than 4–8 h of screen time per day. This approach was based on the observation that unlike cannabis and tobacco use, sedentary behavior and unhealthy food consumption are universal behaviors with which moderate levels of engagement are ubiquitous. When studies reported figures from several different multivariate models, the review included the figures from the final, most stringently adjusted model. Sensitivity analyses attempted to account for the inclusion of studies focusing on specialty samples and disordered behavior patterns.

### Analyses

#### Across studies

The meta-analysis compared mean odds ratios for associations between behaviors and disorders across studies. To compare odds ratio means related to a given unhealthy behavior and a given mental disorder in relation to other such disorders, the data was restructured so as to combine odds ratios for different disorders into a single variable with different corresponding labels. *T*-tests were used for each pair of disorders for cross-sectional and longitudinal (behavior before disorder) studies. With 21 pairs of disorders, 4 behaviors, and 2 analytical approaches, a total of 168 different comparative analyses were performed. To compare odds ratio means between different unhealthy behaviors, findings for each disorder were compared for each pair of behaviors for cross-sectional and longitudinal (behavior before disorder) studies. With seven disorders, six pairs of behaviors, and two analytical approaches, a total of 84 different comparative analyses were performed between studies of different behaviors. It might be noted that none of these comparisons were Bonferroni corrected, which would probably have rendered them all non-significant.

#### Within studies

Some studies reported comparable odds ratios for several different disorders and/or behaviors. In studies of cannabis use that reported figures relating both to psychosis and to other disorders, the review compared the respective odds ratios and classified the figure for psychosis as stronger if it exceeded the other figure by 0.25, as weaker if the other figure exceeded the figure for psychosis by 0.25, and otherwise as being at the same level. Similarly, for studies that provided odds ratios relating to a given disorder for both cannabis/tobacco use and diet quality/sedentary behavior, the review classified either as stronger if it exceeded its counterpart by 0.25, and otherwise as being at the same level.

### Sensitivity analyses

#### Sample groups

The studies included in the review analyzed participant samples ranging from representative samples of the nation's adult population to more narrowly defined groups such as children, older adults, male military conscripts, university students, and patient groups. In order to control for the possibility that narrow samples unduly impacted overall dataset analyses, dichotomous variables for “children” (samples below age 18), “older adults” (samples above age 60), “patient” (all patient or high-risk groups without controls), and “special” (samples consisting of university students or employees, all-male or all-female samples, military service members or veterans, and civil servants) were defined. Subsequently, these variables were combined into a single dichotomous indicator for specialty sample.

#### Behavioral measurement disparities

The studies included in the review spanned behaviors across the risk spectrum, from any (or ever) use of tobacco or weekly fast-food consumption to nicotine use disorder and binge eating disorder. In order to control for the possibility that the disordered behavior measured in some studies unduly impacted overall analyses, a dichotomous “problem behavior” indicator was defined to include behavioral variables including “binge eating disorder,” “emotional eating,” “maladaptive/pathological internet use,” “smartphone addiction,” “Facebook overuse,” “problematic gaming,” “internet addiction,” tobacco/cannabis problem use, abuse, dependence, use disorder, or high-risk use, and smoking during pregnancy. Especially for tobacco, however, it was not clear whether use measured in terms of daily smoking could be meaningfully differentiated from use measured in terms of abuse, dependence etc.

#### Control variables

The studies included in the review used a wide variety of control variables in their statistical analyses. In this review, systematic differences in association strengths related to statistical control for diet quality or sedentary behavior (for tobacco and cannabis analyses), tobacco use (for diet quality, sedentary behavior, or cannabis analyses), and cannabis use (for diet quality, sedentary behavior, or tobacco analyses) were controlled for by the application of dichotomous adjustment variables (0 = no control, 1 = control). Furthermore, dichotomous variables for adjustment for social misery (1 = inclusion of any control variable relating to poverty, education, or unemployment) and psychological trauma (1 = inclusion of any control variable relating to childhood abuse/neglect, bullying victimization, or violence victimization) were defined. Finally, a variable for studies that reported bivariate associations (1 = bivariate) was defined.

It should be noted, however, that some studies analyzed potential covariates on a bivariate basis and only included those that passed a significance threshold of *p* < 0.05 in the final model. This review accepted such variables as having been controlled for, although it should be clear that a covariate may exert meaningful influence on the outcome of a multivariate regression even if its bivariate correlation with the outcome variable is significant only at the 90% (*p* < 0.1) level, for instance.

## Results

### Overall findings

From 3,682 records, 294 were included. Some publications reported figures for several behaviors and methodological approaches (commonly, both cross-sectional and longitudinal), and counting these the review included 97 studies of cannabis use, 132 studies of tobacco use, 88 studies of sedentary behavior, and 47 studies of diet quality. The dataset included a total of 570 associations between behaviors (cannabis use, tobacco use, sedentary behavior, and diet quality) and disorders (psychosis, depression, anxiety, bipolar disorder, personality disorder, ADHD, and PTSD).

#### Sample groups

The review included 320 associations (out of 570) marked as relating to specialty samples. In the full dataset of cross-sectional and longitudinal associations, the specialty sample indicator was not significantly correlated with odds ratios for any disorder or behavior. Specialty samples were most commonly used in studies of diet quality and sedentary behavior (69%) and least commonly used in cannabis studies (44%). As a sensitivity test, the analyses reported below were repeated with studies using specialty samples removed from the dataset. This new set of analyses identified marginally significant differences (*t* = 2.30, df = 5.76, two-sided *p* = 0.06) for PTSD between cannabis use and tobacco use, with the former having higher ORs.

#### Behavioral measurement disparities

The review included 101 associations (out of 570) marked as relating to disordered or problematic behavior. In the full dataset of cross-sectional and longitudinal associations, the problem behavior indicator was significantly associated with odds ratios for depression (*r* = 0.15, *p* = 0.015, *N* = 251) and ADHD (*r* = 0.31, *p* = 0.021, *N* = 55). The inclusion of studies investigating problem behaviors may thus have served to increase association strengths for these disorders. Across disorders, the indicator for problem behavior was significantly correlated with the odds ratio strength in non-tobacco associations (*r* = 0.12, *p* = 0.021, *N* = 366) but not in associations with tobacco use (*r* = 0.08, *p* = 0.252, *N* = 204). This finding supports the notion that tobacco use measured in terms of daily smoking cannot necessarily be differentiated from use measured in terms of abuse or dependence. Problem behaviors were most commonly studied in relation to cannabis use (29%) and least commonly in relation to diet quality and sedentary behavior (10%). As a sensitivity test, the analyses reported below were repeated with studies investigating disordered or problematic behavior removed from the dataset. This new set of analyses identified significant differences (*t* = 2.31, df = 8.38, two-sided *p* < 0.05) for PTSD between cannabis use and tobacco use, as well as between cannabis use and sedentary behavior (*t* = 2.32, df = 8.13, two-sided *p* < 0.05), with ORs for cannabis in both cases being higher. Furthermore, these analyses identified marginally significant differences (*t* = 1.93, df = 32, two-sided *p* = 0.06) between cannabis-psychosis and cannabis-PTSD, with the latter having higher ORs. Finally, the review included a few studies that investigated the association between depression and secondhand smoking (*N* = 5) and e-cigarette use (*N* = 6). These studies did not diverge substantially from other tobacco studies in terms of their association strengths with mental disorders.

#### Control variables

Some studies reported bivariate associations (33 out of 570), generally because their main focus lay elsewhere and the relationships investigated in this review were only a secondary concern in their analysis. In the full dataset, the bivariate indicator was significantly associated with odds ratios for anxiety (*r* = 0.19, *p* = 0.043, *N* = 112) and ADHD (*r* = 0.33, *p* = 0.013, *N* = 55). The inclusion of studies reporting bivariate associations may thus have served to increase association strengths for these disorders. Across disorders and behaviors, the bivariate indicator was marginally correlated with odds ratios (*r* = 0.08, *p* = 0.064, *N* = 570). Bivariate findings were most commonly reported in tobacco studies (8%) and least commonly in studies of sedentary behavior and diet quality (4%). As a sensitivity test, the analyses reported below were repeated with studies reporting bivariate associations removed from the dataset. This new set of analyses identified marginally significant differences (*t* = 1.96, df = 18.57, two-sided *p* = 0.07) for PTSD between cannabis use and sedentary behavior, with the former having higher ORs.

Overall, 51 (out of 570) associations were adjusted for psychological trauma, and the indicator for trauma adjustment was marginally significant for psychosis (*r* = −0.25, *p* = 0.056, *N* = 61). Across disorders and behaviors, the indicator was marginally correlated with odds ratios (*r* = −0.07, *p* = 0.103, *N* = 570), and such adjustment was most commonly employed in cannabis studies (14%) and least commonly in studies of diet quality (2%). By contrast, 441 (out of 570) associations were adjusted for social misery, and such adjustment was most commonly employed in studies of diet quality (82%) and least in studies of cannabis and tobacco (76%). Across disorders and behaviors, the indicator for misery adjustment trended weakly in a negative direction (*r* = −0.06, *p* = 0.151, *N* = 570) but was marginally correlated with odds ratios in cannabis studies (*r* = −0.13, *p* = 0.093, *N* = 178).

Only 1% of cannabis studies and 14% of tobacco studies controlled for diet quality and/or sedentary behavior, while studies of the latter behaviors often controlled for tobacco (55%) but rarely for cannabis (8%). Furthermore, 35% of cannabis studies controlled for tobacco while 24% of tobacco studies controlled for cannabis. With regard to specific disorders, the indicator for cannabis adjustment was significant only in (non-cannabis) studies of psychosis (*r* = −0.55, *p* < 0.001, *N* = 32) while tobacco adjustment was significant in (non-tobacco) studies of anxiety (*r* = −0.24, *p* = 0.033, *N* = 80) and marginally of depression (*r* = −0.15, *p* = 0.059, *N* = 163). Across disorders, tobacco adjustment was significantly correlated with odds ratios in non-tobacco studies (*r* = −0.19, *p* < 0.001, *N* = 366) and cannabis adjustment was significantly correlated with odds ratios in non-cannabis studies (*r* = −0.13, *p* = 0.010, *N* = 392). The indicator for adjustment for diet quality and/or sedentary behavior was nowhere significant.

In exploratory linear regression analyses, the set of study quality variables (sample size, specialty sample, disordered behavior, and control variables) explained 2.7% of the variance in odds ratios in the overall dataset and 12.4% of the variance in longitudinal studies. For cross-sectional studies of individual behaviors, regression models explained between 9.0% (tobacco use) and 27.5% (poor diet quality) of the variance in odds ratios, and for individual disorders between 1.4% (personality disorder) and 37.5% (bipolar disorder). See Supplementary Appendix B for the specification of these models. In these models, disordered behavior commonly reached significance as a positive influence on odds ratios, indicating that studies investigating such behaviors tended to find higher risk for mental disorders. In the full dataset, higher sample size reduced odds ratios, indicating that large-*N* studies tended to obtain more moderate risks; this was an issue especially for longitudinal (behavior before disorder) studies. In studies of cannabis and tobacco use, adjustment for its counterpart was a highly significant negative influence on odds ratios.

### Findings for individual disorders

#### Psychosis

This review included 45 cross-sectional and 16 longitudinal figures for the association between unhealthy behaviors and psychosis (Tables 1a, 1b). The cross-sectional studies provide convincing evidence for associations between psychosis and cannabis and tobacco use and some evidence for associations with sedentary behavior and diet quality. The longitudinal studies provide some evidence that cannabis and tobacco use may lead to subsequent psychosis and more tenuous evidence that psychosis may lead to subsequent cannabis and tobacco use.

**Table 1a:** Cross-sectional psychosis studies (*N* = 45).

	***N***	**Area**	**Sample**	**Exposure**	**OR**	**Covariate adjustments**				
**Cannabis (*****N*** = **20)**						**SD**	**TO**	**MI**	**TR**	**BV**
Livne et al. (170)	79,402	USA	Adults 18+	Weekly to daily	3.95		•	•		
Davies et al. (171)	2,475	UK	Adults 18–20	Problem	3.64			•		
McMahon et al. (120)	928	IRL	Schoolchildren	Past year	3.52					ø
Bhavsar et al. (172)	1,680	UK	Adults 16–90	Weekly to daily	3.00					ø
Bassir Nia et al. (173)	594	USA	Adult patients	Any non-synth.	1.87					
Padwa et al. (151)	8,940	USA	Adult patients and contr.	Any	1.69		•			
Wainberg et al. (174)	109,308	UK	Adults 40–69	Weekly	1.69			•		
Pardo et al. (175)	152	SPA	Child patients and contr.	Non-daily	1.65					
Campbell et al. (176)	101,405	USA	Patients 18+	Disorder	1.62		•	•		
Ferraro et al. (177)	2,261	Int.	Adult patients and contr.	Current	1.61					
Degenhardt et al. (121)	514	AUS	Adolescent offenders	Weekly	1.50		•	•	•	
Corsi-Zuelli et al. (178)	409	BRA	Patients and controls	<daily	1.48		•	•		
Carney et al. (179)	731	AUS	Help-seeking youth	Current	1.40					ø
Di Forti et al. (180)	2,138	Int.	Adult patients and contr.	Weekly >1	1.40		•	•		
McGuckin et al. (181)	81,809	CAN	Adult patients	Past 30 days	1.31		•	•	•	
Hines et al. (182)	1,087	UK	Adults	High potency	1.29			•		
Chan et al. (183)	181,870	Int.	Adults 18+	Potent herb	1.28			•		
Di Forti et al. (184)	780	UK	Adult patients and contr.	Weekly	1.04		•	•		
Bassir Nia et al. (185)	983	USA	Adult patients	Any non-synth.	1.02					
Sideli et al. (140)	445	UK	Patients and controls	Non-daily	0.97			•	•	
**Tobacco (*****N*** = **19)**						**SD**	**CA**	**MI**	**TR**	**BV**
Molla et al. (186)	422	ETH	Adult patients	Dependence	5.26			•		
McMahon et al. (120)	928	IRL	Schoolchildren	Past year	3.61					ø
Dickerson et al. (187)	1,938	USA	Adult patients and contr.	Current	3.58			•		
Ferraro et al. (177)	2,261	Int.	Adult patients and contr.	Current	3.47					
Zolezzi et al. (188)	196	QAT	Patients 15+	Current	2.48					ø
Werneck et al. (189)	60,202	BRA	Adult patients and contr.	Current	2.39			•		
Wolfe et al. (190)	930	USA	University students	Current	2.31		•			
Clark et al. (191)	421	AUS	Patients 14–25	Current	2.17		•			
Mustonen et al. (192)	5,926	FIN	Adolescents	Daily	2.17					ø
Yilmaz Kafali et al. (193)	684	TUR	Adol. Patients and controls	Past 30 days	2.11		•			
Li et al. (51)	1,102	CHN	Adult patients	Daily	2.00			•		
Davies et al. (171)	2,491	UK	Adolescence->adulthood	Weekly +	1.68			•		
Chang et al. (122)	5,719	CHN	Adults 16–75	Current	1.65		•	•	•	
Carney et al. (179)	731	AUS	Help-seeking youth	Daily	1.50					ø
Bhavsar et al. (172)	1,680	UK	Adults 16–90	Daily	1.47		•	•		
Mallet et al. (194)	34,653	USA	Adults	Current	1.36		•	•		
Fang et al. (195)	469	CHN	Adult patients and contr.	Any	1.32					
Degenhardt et al. (121)	514	AUS	Adolescent offenders	Daily	1.10		•	•	•	
Bourbon et al. (125)	10,985	FRA	University students	Daily	1.01		•	•	•	
**Sedentary behavior (*****N*** = **4)**						**TO**	**CA**	**MI**	**TR**	**BV**
Werneck et al. (189)	60,202	BRA	Adult patients and contr.	Tv >5 h/day	3.84			•		
McMahon et al. (120)	749	IRL	Schoolchildren	Pathological	2.70					
Kim et al. (196)	6,510	KOR	Adults 18–64	Addiction	2.32			•		
Zhang et al. (197)	7,121	CHN	Adults 18–81	Tv >3 h/day	1.61	•		•		
**Diet quality (*****N*** = **2)**										
Werneck et al. (189)	60,202	BRA	Adult patients and contr.	Daily sweets	2.36			•		
Mutiso et al. (198)	9,742	KEN	High school students	Binge eating	1.41	•	•	•		
	**Median**	**Unweighted mean (SD)**		**Weighted mean**						
Cannabis	1.56	1.85 (0.91)		1.82						
Tobacco	2.11	2.24 (1.07)		1.97						
Sedentary behavior	2.51	2.62 (0.93)		3.48						
Diet quality	1.88	1.88 (0.67)		2.23						

OR, odds ratio; SD, sedentary behavior and diet quality; TO, tobacco; CA, cannabis; MI, social misery; TR, psychological trauma; BV, bivariate (unadjusted).

**Table 1b:** Longitudinal psychosis studies (*N* = 16).

**Longitudinal: behavior before disorder (*****N*** = **9)**					**Covariate adjustments**					
**Cannabis (*****N*** = **5)**	***N***	**Area**	**Sample**	**Exposure**	**OR**	**SD**	**TO**	**MI**	**TR**	**BV**
van Os et al. (199)	6,615	Int.	Adolescents and adults	>5 times	7.03			•		
Jones et al. (200)	5,300	UK	Adolescence->adulthood	Any	3.12			•	•	
Bechtold et al. (201)	908	USA	Adolescent boys	Weekly	1.52					ø
Degenhardt et al. (202)	30,902	Int.	Adults	Any	1.00		•			
Ryan et al. (203)	11,755	USA	Adults 18–34	Before age 16	0.96		•	•	•	
**Tobacco (*****N*** = **4)**						**SD**	**CA**	**MI**	**TR**	**BV**
Rognli et al. (204)	2,602	NOR	Adolescence->adulthood	Daily, low dep.	3.79			•		
Ryan et al. (203)	11,755	USA	Adults 18–34	Before age 16	1.60		•	•	•	
Degenhardt et al. (202)	30,902	Int.	Adults	Any	1.10		•			
Jones et al. (200)	5,300	UK	Adolescence->adulthood	Any	0.96		•	•	•	
	**Median**	**Unweighted mean (SD)**		**Weighted mean**						
Cannabis	1.52	2.73 (2.56)		1.92						
Tobacco	1.35	1.86 (1.31)		1.34						
**Longitudinal: disorder before behavior (*****N*** = **7)**					**Covariate adjustments**					
**Cannabis (*****N*** = **4)**	***N***	**Area**	**Sample**	**Outcome**	**OR**	**SD**	**TO**	**MI**	**TR**	**BV**
Jones et al. (200)	5,300	UK	Adolescence->adulthood	Any use	1.50			•	•	
Degenhardt et al. (202)	30,902	Int.	Adults	Any use	1.30		•			
van Os et al. (199)	6,466	Int.	Adolescents and adults	>5 times	0.59			•		
Davies et al. (171)	2,602	UK	Adolescence->adulthood	Problem use	0.54			•		
**Tobacco (*****N*** = **3)**						**SD**	**CA**	**MI**	**TR**	**BV**
Jones et al. (200)	5,300	UK	Adolescence->adulthood	Any use	1.43		•	•	•	
Davies et al. (171)	2,627	UK	Adolescence->adulthood	Weekly +	1.11			•		
Degenhardt et al. (202)	30,902	Int.	Adults	Any use	1.10		•			
	**Median**	**Unweighted mean (SD)**		**Weighted mean**						
Cannabis	0.95	0.98 (0.49)	1.18					
Tobacco	1.11	1.21 (0.19)	1.15					

OR, odds ratio; SD, sedentary behavior and diet quality; TO, tobacco; CA, cannabis; MI, social misery; TR, psychological trauma; BV, bivariate (unadjusted).

#### Depression

The present review included 208 cross-sectional and 43 longitudinal figures for the association between unhealthy behaviors and depression (Tables 2a, 2b). The cross-sectional studies provide convincing evidence for associations between depression and cannabis, tobacco, sedentary behavior, and diet quality. The longitudinal studies provide some evidence that sedentary behavior and use of cannabis and tobacco may lead to subsequent depression and that depression may lead to subsequent cannabis and tobacco use. There was very limited longitudinal evidence related to diet quality.

**Table 2a:** Cross-sectional depression studies (*N* = 208).

	***N***	**Area**	**Sample**	**Exposure**	**OR**	**Covariate adjustments**				
**Cannabis (*****N*** = **43)**						**SD**	**TO**	**MI**	**TR**	**BV**
Metrik et al. (205)	301	USA	Male military veterans	Weekly 2+	4.88					
Halladay et al. (206)	43,466	CAN	Persons 15+	Weekly >1	3.91					
Wahby et al. (207)	302	CAN	Patients 18+	Current	3.90			•		
Davies et al. (171)	2,475	UK	Adults 18–20	Problem	3.55			•		
Magklara et al. (153)	2,427	GRC	Adolescents	Any	3.48					
Risal et al. (208)	2,100	NPL	Adults 18–65	Any	3.10					ø
Porras-Segovia et al. (114)	810	ESP	Adults 18–80	Any	2.95				•	
Hasin et al. (136)	36,309	USA	Adults 18+	Disorder	2.80			•		
Hasin and Walsh (20)	36,309	USA	Adults 18+	Disorder	2.60			•		
Padwa et al. (151)	8,940	USA	Adult patients and contr.	Any	2.46		•			
Gorfinkel et al. (209)	16,216	USA	Adults 20–59	Daily or near	2.29			•		
Carrà et al. (210)	527,446	USA	Adults 18+	Weekly to daily	2.28					
Cougle et al. (211)	43,093	USA	Adults	Weekly	2.27			•		
Livne et al. (169)	36,309	USA	Adults 18+	Non-CUD	2.25			•		
Young-Wolff et al. (123)	196,022	USA	Pregnant women	During pregnancy	2.25			•		
Pacek et al. (212)	728,691	USA	Persons 12+	Past 30 days	2.17			•		
Cougle et al. (213)	43,093	USA	Adults	Weekly	2.03			•		
Wu et al. (214)	10,734	USA	Patients 18+	Disorder	1.74			•		
Hill et al. (215)	3,157	USA	Military veterans	Non-CUD	1.65			•		
Keith et al. (216)	1,776	USA	University students	Weekly 1–2	1.60					
Mannes et al. (217)	932	USA	Adult patients	Past year	1.50			•		
Chadi et al. (218)	26,821	USA	Adolescents	Past 30 days	1.49		•			
Halladay et al. (219)	43,466	CAN	Persons 15–60	Monthly +	1.48					
Butler et al. (220)	6,550	CAN	Adolescents	Any	1.40		•	•		
Estévez et al. (221)	5,677	SWI	Male military conscripts	Weekly >1	1.35			•		
Leventhal et al. (222)	3,177	USA	Adolescents	Past 30 days	1.31		•	•		
Hines et al. (182)	1,087	UK	Adults	High potency	1.28			•		
Prestage et al. (223)	3,017	AUS	Gay and bisexual men	Past 6 months	1.27					ø
Kerridge et al. (224)	36,309	USA	Adults 18+	Disorder	1.25		•	•		
Rubenstein et al. (225)	262	USA	African americans 18+	Any	1.22		•	•		
Chan et al. (183)	181,870	Int.	Adults 18+	Potent herb	1.18			•		
Seaman et al. (226)	2,555	USA	Adults 21–30	Past 30 days	1.18		•	•		
Gukasyan and Strain (227)	87,952	USA	Adolescents 12–17	Weekly +	1.16			•		
Wang and Peiper (228)	13,526	USA	High school students	Past 30 days	1.15	•	•		•	
Hill et al. (229)	4,069	USA	Military veterans	Past 6 months	1.12			•	•	
Campbell et al. (176)	101,405	USA	Patients 18+	Disorder	1.08		•	•		
Lekoubou et al. (230)	400,391	USA	Patients 18+	Disorder	1.07			•		
Bonsaksen et al. (231)	4,527	NOR	Adults 18+	Any	1.06			•		
Fink et al. (232)	392	USA	Adult patients	Mod. Disorder	1.05			•		
Thompson et al. (233)	662	CAN	Adolescence->adulthood	>1/week	0.93		•	•		
**Cannabis (*****N*** = **43)**						**SD**	**TO**	**MI**	**TR**	**BV**
Welsh et al. (234)	483	USA	Patients 11–24	Disorder	0.78					
Tiburcio Sainz et al. (235)	710	MEX	University students	Mod-high risk	0.73		•	•		
Gaete et al. (236)	935	CHL	Young offenders 14–23	Any	0.60		•	•	•	
**Tobacco (*****N*** = **71)**						**SD**	**CA**	**MI**	**TR**	**BV**
Raffetti et al. (237)	3,062	SWE	Adolescents	Past 30 days	3.90			•		
Farrell et al. (238)	4,961	USA	Adults 18+	E-cig.	3.61			•		
Klein et al. (239)	11,785	USA	Children 9–12	Any	3.37			•		
Mossie et al. (128)	590	ETH	Adults 18+	Past 30 days	3.15			•		
Shahwan et al. (134)	6,126	SGP	Adults 18+	Dependence	3.00					
Albasara et al. (130)	342	SAU	Adult patients	Any	2.99	•		•		
Mohammadi et al. (240)	299	AFG	Healthcare work. 18–64	Current	2.96			•		
Conti et al. (241)	220	BRA	Men 18–65	Any	2.81			•		
Kelishadi et al. (242)	13,486	IRN	Children 6–18	Daily	2.65			•		
Bernard et al. (243)	334	Int.	Patients 50+	Any	2.60		•	•	•	
Wang et al. (244)	10,349	USA	Adults 20+	>100 cig.	2.56	•		•		
Rahe et al. (245)	1,420	GER	Patients and controls	Current	2.39			•		
Ma et al. (246)	3,787	SWE	Adolescents	Monthly	2.34	•		•		
Islam et al. (129)	600	BGD	Older adults P and C	Current	2.33	•		•		
Weinberger et al. (247)	496,805	USA	Persons 12+	Non-daily	2.22			•		
Davies et al. (171)	2,491	UK	Adults 18–20	Weekly +	2.19			•		
van Binnendijk et al. (248)	22,471	NLD	Adults 18–70	Dependence	2.18			•		
Chido-Amajuoyi et al. (249)	2,034	USA	Adults 18+	Past 30 days	2.12			•		
Magklara et al. (153)	2,427	GRC	Adolescents	Daily	2.05					
Ye et al. (250)	1,280	KOR	Women 40–60	Secondhand	2.04			•		
Lee and Lee (251)	62,276	CHN	Adolescents	Past 30 days	2.04			•		
López-Sánchez et al. (133)	4,157	ESP	Adult patients	Current	2.03	•		•		
Cougle et al. (211)	43,093	USA	Adults	Weekly	2.01			•		
Islam et al. (252)	563	BGD	Adolescents	Current	2.00	•		•		
Nam et al. (131)	4,145	KOR	Adults 20+	Current	1.94	•		•		
Matcham et al. (253)	7,878	USA	Adult patients	Current	1.93					
Gorfinkel et al. (254)	32,636	UK	Adolescents	Past 30 days	1.93		•	•		
Melin et al. (255)	1,027	SWE	Adult patients	Current	1.90					ø
Chen et al. (256)	17,837	TWN	Persons 12–64	Any	1.75		•	•		
Wiernik et al. (257)	35,337	CHN	Adults 18–69	Current e-cig	1.73			•		
Luo et al. (258)	49,317	FRA	Older adults	Current	1.73					ø
Cougle et al. (213)	43,093	USA	Adults	Daily	1.69			•		
Formagini et al. (259)	48,282	BRA	Adults 18+	Daily	1.66			•		
Patanavanich et al. (260)	4,237	THA	Adolescents	Any e-cig.	1.66			•	•	
Masana et al. (261)	2,718	Int.	Older adults	Current	1.60	•		•		
Bandiera et al. (262)	5,438	USA	University students	Past 30 days	1.58					
Prestage et al. (223)	3,017	AUS	Gay and bisexual men	Daily	1.54		•	•		
**Tobacco (*****N*** = **71)**						**SD**	**CA**	**MI**	**TR**	**BV**
Kim (263)	3,700	KOR	Older adults 65+	Current	1.53	•		•		
Kim et al. (264)	366,405	KOR	Adolescents	Any	1.51	•		•		
Risal et al. (208)	2,100	NPL	Adults 18–65	Any	1.50					ø
Seaman et al. (226)	2,555	USA	Adults 21–30	Past 30 days	1.49		•	•		
Liu et al. (265)	1,300	USA	Adults 20–80	Daily	1.47	•		•		
Patten et al. (266)	184,305	CAN	Adults	Secondhand	1.40			•		
Porras-Segovia et al. (114)	810	ESP	Adults 18–80	Dependence	1.40					ø
Kelishadi et al. (242)	13,486	IRN	Children 6–18	Secondhand	1.37			•		
Chadi et al. (218)	26,821	USA	Adolescents	E-cig.	1.37					
Werneck et al. (189)	60,202	BRA	Adult patients and contr.	Current	1.37			•		
Clyde et al. (267)	1,614	CAN	Adult patients	Daily	1.34			•		
Chen et al. (268)	2,590	TWN	Women 45–55	Secondhand	1.33			•		
Liu et al. (269)	5,965	CHN	Men 40–79	Current	1.32					
Okunna (135)	277,034	USA	Adults 18+	>100 cig.	1.30		•	•		
Salimi et al. (270)	29,654	USA	Pregnant women	Postpartum use	1.28			•		
Chou et al. (271)	36,309	USA	Adults 18+	Disorder	1.26			•		
Peltzer and Pengpid (272)	20,222	Int.	University students	Current	1.25	•		•	•	
Wang and Peiper (228)	13,526	USA	High school students	Past 30 days	1.25	•	•		•	
Estévez et al. (221)	5,677	SWI	Male military conscripts	Daily	1.24			•		
Bourbon et al. (125)	10,985	FRA	University students	Daily	1.24		•	•	•	
Sumbe et al. (273)	2,439	USA	Adolescents and adults	Past 30 days	1.23		•	•		
Kastaun et al. (274)	11,937	GER	Adults 18+	Current	1.20			•		
Ellis et al. (275)	3,468	USA	Adults	Any	1.17			•		
Tiburcio Sainz et al. (235)	710	MEX	University students	Mod-high risk	1.17		•	•		
Pengpid and Peltzer (276)	4,782	ZAF	Adults 40+	Current	1.16	•		•		
Assari et al. (277)	740	USA	African Am. adults 55+	Current	1.13			•		
Li et al. (278)	1,504	CHN	University students	Current	1.06					ø
Sawchuk et al. (279)	2,774	USA	American Indians 15–54	>100 cig.	1.01			•		
Chou et al. (280)	36,309	USA	Adults 18+	Any e-cig.	1.00		•	•		
Hruby et al. (281)	12,708	USA	Military service members	Past 30 days	0.93	•	•	•		
Mannes et al. (217)	932	USA	Adult patients	Past 30 days	0.86			•		
Zhu et al. (282)	4,043	CHN	Adult patients	Current	0.77	•		•		
Zolezzi et al. (188)	196	QAT	Patients 15+	Current	0.70					ø
Welsh et al. (234)	483	USA	Patients 11–24	Disorder	0.57					
**Sedentary behavior (*****N*** = **61)**						**TO**	**CA**	**MI**	**TR**	**BV**
Humer et al. (283)	6,703	AUT	Adolescents	Phone >8 h/day	6.79					
Xie et al. (284)	2,134	CHN	University students	Worst quartile	5.76	•		•		
Zhu et al. (285)	4,043	CHN	Adult patients	Sedentary	4.73	•		•		
Galán-Arroyo et al. (286)	17,141	ESP	Adults 18–69	Sedentary	4.32					
Xu et al. (287)	480	CHN	University students	Worst half	4.31			•		
**Sedentary behavior (*****N*** = **61)**						**TO**	**CA**	**MI**	**TR**	**BV**
Wang et al. (56)	2,679	CHN	Older adults 60+	TV >3 h/day	3.59	•		•		
Adamson et al. (288)	3,045	USA	Adults 18+	Worst quartile	3.47	•		•		
Luo et al. (258)	49,317	CHN	Older adults	>6 h/day	3.16					ø
Pengpid and Peltzer (276)	4,782	ZAF	Adults 40+	>11 h/day	3.00	•		•		
Jiang et al. (289)	28,298	CHN	University students	Sedentary	2.94	•		•		
Liu et al. (265)	1,300	USA	Adults 20–80	Low PA	2.76	•		•		
Albasara et al. (130)	342	SAU	Adult patients	Low PA	2.65	•		•		
Kim et al. (196)	6,510	KOR	Adults 18–64	Addiction	2.65			•		
Vadlin et al. (290)	2,110	SWE	Patients and controls	Problematic gaming	2.47				•	
Madhav et al. (132)	3,201	USA	Adults 20+	scr. t. >6 h/day	2.35			•		
Alageel et al. (291)	506	Int.	University students	Addiction	2.11	•				
Werneck et al. (292)	60,202	BRA	Adults 18+	TV >5 h/day	2.10	•		•		
Vancampfort et al. (293)	4,082	Int.	Adult patients	>8 h/day	1.99	•		•		
Yu et al. (294)	18,994	CHN	Adults	TV >10 h/day	1.95	•		•		
Trinh et al. (295)	2,660	CAN	Adolescents	scr. t. >2 h/day	1.92			•		
Vancampfort et al. (296)	6,903	IRL	Adults 50+	>8 h/day	1.88	•		•		
Wu et al. (297)	4,747	CHN	University students	scr. t. >2 h/day	1.86			•		
da Costa et al. (298)	293	BRA	Older adults 60+	>4.5 h/day	1.81			•		
Lazarevich et al. (299)	615	MEX	Female uni. Students	PA < 75 min/week	1.80					
Melin et al. (255)	1,027	SWE	Adult patients	PA < 1/week	1.80					ø
Liu et al. (300)	13,659	CHN	Adolescents	Games >2 h/day	1.78	•		•		
Jensen-Otsu and Austin (301)	3,039	USA	Adults 20–74	Games >2 h/day	1.77	•		•		
Zhu et al. (282)	4,043	CHN	Adult patients	Low PA	1.74	•		•		
Cho et al. (302)	15,146	KOR	Older adults 60+	Low PA	1.73	•		•		
Zhang et al. (303)	27,723	CHN	University students	scr. t. >4 h/day	1.72					
Nam et al. (131)	4,145	KOR	Adults 20+	>10 h/day	1.71	•		•		
Rahe et al. (245)	1,420	GER	Patients and controls	Low PA	1.71			•		
da Costa et al. (304)	610	BRA	Adolescents	SM >4 h/day	1.67			•		
Silva et al. (305)	88,509	BRA	Adults 18+	TV >6 h/day	1.67			•		
Lu et al. (306)	965	CHN	Adolescents	>4 h/day	1.65			•		
Wang and Peiper (228)	13,526	USA	High school students	scr. t. >3 h/day	1.61	•	•		•	
Ma et al. (246)	3,787	SWE	Adolescents	PA < 1/month	1.58	•		•		
Wu et al. (307)	2,521	CHN	University students	ST >2 h/day	1.58	•		•		
Zhou et al. (308)	584	CHN	University students	scr. t. >1.4 h/day	1.54	•		•		
Vancampfort et al. (309)	67,077	Int.	Adolescents	>8 h/day	1.53			•		
Hanna et al. (310)	479	QAT	University employees	>10 h/day	1.41					
Peltzer and Pengpid (272)	20,222	Int.	University students	Internet 6+ h/day	1.40	•		•	•	
Schuch et al. (311)	937	BRA	Adults 18+	>10 h/day	1.40	•		•		
López-Sánchez et al. (133)	4,157	ESP	Adult patients	Inactivity	1.37	•		•		
Kim and Han (312)	54,603	KOR	Adolescents	Phone >4 h/day	1.37			•		
Werneck et al. (189)	60,202	BRA	Adult pat. and contr.	TV >5 h/day	1.34			•		
**Sedentary behavior (*****N*** = **61)**						**TO**	**CA**	**MI**	**TR**	**BV**
Apriliyasari et al. (313)	3,234	IDN	Adult patients	Low PA	1.32	•		•		
Carriedo et al. (314)	483	ESP	Older adults 60–92	Low PA	1.29					
Souza et al. (315)	6,924	BRA	Older adults 60+	PA < 1/week	1.28	•		•		
Liao et al. (316)	2,914	JPN	Adults 20–59	SB >6 h/day	1.25			•		
Pengpid and Peltzer (317)	72,262	IND	Adults 45+	Low PA	1.24	•		•		
Masana et al. (261)	2,718	Int.	Older adults	Current	1.23	•		•		
Kim (263)	3,700	KOR	Older adults 65+	Low PA	1.19	•		•		
Pengpid and Peltzer (318)	3,201	Int.	Adult patients	>8 h/day	1.19	•		•		
Hruby et al. (281)	12,708	USA	Military service members	PA < 75 min/week	1.17	•	•	•		
Taheri et al. (319)	13,486	IRN	Children 6–18	scr. t. >2 h/day	1.15	•		•		
Islam et al. (252)	563	BGD	Adolescents	Low PA	1.10					ø
Zhang et al. (197)	7,121	CHN	Adults 18–81	TV >3 h/day	1.07	•		•		
Kim et al. (264)	366,405	KOR	Adolescents	Worst quartile	1.07	•		•		
Draper et al. (320)	1,719	ZAF	Women 18–26	Low PA	0.84					
Padmapriya et al. (321)	863	SGP	Pregnant women 18+	TV >2 h/day	0.75	•		•		
**Diet quality (*****N*** = **33)**						**TO**	**CA**	**MI**	**TR**	**BV**
Sze et al. (152)	424	CHN	University students	Emotional eating	28.19			•		
ElBarazi and Tikamdas (322)	509	EGY	University students	Daily junk food	7.90					ø
Khosravi et al. (323)	330	IRN	Adult patients and contr.	Worst quartile	3.62			•		
Islam et al. (129)	600	BGD	Older adults P and C	Poor diet	3.44	•		•		
Gomes et al. (324)	1,378	BRA	Older adults 60+	Worst tertile	2.96	•		•		
Mutiso et al. (198)	9,742	KEN	High school students	Binge eating	2.48	•	•	•		
Liu et al. (325)	1,311	CHN	Children 7–17	Daily soda	2.28			•		
Lazarevich et al. (299)	615	MEX	Female uni. Students	Fast f. >1/week	2.08					
Hong and Peltzer (326)	65,212	KOR	Adolescents	Soda daily 3+	2.07	•		•		
Liu et al. (265)	1,300	USA	Adults 20–80	Poor diet	2.07	•		•		
Kim et al. (327)	849	KOR	Adolescent girls	Worst tertile	2.03					
Yu et al. (328)	3,667	CHN	Adults	Soda >3/w	2.00	•		•		
Zhang et al. (303)	27,723	CHN	University students	Soda >4/day	1.82					
Liu et al. (329)	906	CHN	Postmenopaus. Women	Worst tertile	1.79			•		
Vicente et al. (330)	406	BRA	Adult patients	High soda	1.73			•		
Sangsefidi et al. (331)	9,965	IRN	Adults 20–70	Fast food 1/week	1.61	•		•		
Kim (263)	3,700	KOR	Older adults 65+	Poor diet	1.56	•		•		
Kim et al. (332)	5,465	KOR	Adults 20+	Daily soda	1.54	•		•		
Kang et al. (333)	7,446	KOR	Adults 18–65	Worst quartile	1.43	•		•		
Park et al. (334)	65,528	KOR	Adolescents	Fast f. >2/week	1.42			•		
Ra (335)	24,006	KOR	Adolescents	Worst tertile	1.38	•	•	•		
Zheng et al. (336)	13,637	USA	Adults 20+	Worst quartile	1.34	•		•		
Werneck et al. (189)	60,202	BRA	Adult patients and contr.	Daily sweets	1.34			•		
Xia et al. (337)	2,702	CHN	Adult patients and contr.	Worst quartile	1.33	•		•		
**Diet quality (*****N*** = **33)**						**TO**	**CA**	**MI**	**TR**	**BV**
Tran et al. (338)	3,670	FRA	University students	Junk food	1.30	•	•	•		
Miller et al. (339)	3,430	AUS	Adults 18+	Daily soda	1.30			•		
Nouri Saeidlou et al. (340)	510	IRN	Adult female P and C	Western diet	1.29	•		•		
Yim et al. (341)	187,622	KOR	Adolescents	Fast food 1/week	1.20			•		
Wang et al. (244)	10,349	USA	Adults 20+	Poor diet	1.18	•		•		
Knüppel et al. (342)	8,087	UK	Civil servants 35–55	Worst tertile	1.08	•		•		
Hall et al. (343)	444	MEX	University students	Worst half	0.99	•				
Rahe et al. (245)	1,420	GER	Patients and controls	Worst half	0.98			•		
Hosseinzadeh et al. (344)	3,846	IRN	Adults 20–55	Worst quintile	0.94	•		•		
	**Median**	**Unweighted mean (SD)**		**Weighted mean**						
Cannabis	1.49	1.88 (0.99)		1.89						
Tobacco	1.58	1.77 (0.70)		1.70						
Sedentary behavior	1.71	2.04 (1.16)		1.62						
Diet quality	1.56	2.72 (4.74)		1.49						

OR, odds ratio; SD, sedentary behavior and diet quality; TO, tobacco; CA, cannabis; MI, social misery; TR, psychological trauma; BV, bivariate (unadjusted); PA, physical activity; SB, sedentary behavior; SM, social media.

**Table 2b:** Longitudinal depression studies (*N* = 43).

**Longitudinal: behavior before disorder (*****N*** = **30)**					**Covariate adjustments**					
**Cannabis (*****N*** = **7)**	***N***	**Area**	**Sample**	**Exposure**	**OR**	**SD**	**TO**	**MI**	**TR**	**BV**
Matta et al. (150)	37,192	FRA	Adults 18–69	Weekly +	1.73					
Hengartner et al. (345)	591	SWI	Adolescence->adulthood	Adolescence	1.72			•		
Agrawal et al. (346)	13,986	AUS	Twin pairs	>100 times	1.53		•		•	
Cougle et al. (211)	34,653	USA	Adults	Weekly	1.07			•		
Copeland et al. (141)	1,420	USA	Childhood->adulthood	Cumul. daily	1.00		•	•	•	
Blanco et al. (347)	34,653	USA	Adults 18+	Past year	0.90		•	•	•	
Feingold et al. (348)	34,653	USA	Adults 18+	Weekly +	0.67		•	•		
**Tobacco (*****N*** = **12)**						**SD**	**CA**	**MI**	**TR**	**BV**
Bakhshaie et al. (349)	2,101	USA	Adults	Daily	2.30		•			
Raffetti et al. (237)	3,139	SWE	Adolescents	Past 30 days	2.00			•		
Rognli et al. (204)	2,602	NOR	Adolescence->adulthood	Low dep.	1.91			•		
Zhang et al. (350)	1,196	GER	Women 18–25	Current	1.55	•				
Tsutsumimoto et al. (351)	3,066	JPN	Older adults 65+	Current	1.52	•		•		
Matta et al. (150)	37,192	FRA	Adults 18–69	1–19/day	1.48					
Cabello et al. (352)	7,908	Int.	Adults 18–49 and 50+	Daily	1.46	•		•		
Clyde et al. (267)	1,614	CAN	Adult patients	Daily	1.33			•		
Cougle et al. (211)	34,653	USA	Adults	Weekly	1.31			•		
Song et al. (353)	8,842	CHN	Pregnant women	Secondhand	1.24			•		
Song et al. (353)	8,842	CHN	Pregnant women	Daily	1.20			•		
Bolstad et al. (354)	7,660	FIN	Adolescence->adulthood	Daily	1.07		•	•		
**Sedentary behavior (*****N*** = **10)**						**TO**	**CA**	**MI**	**TR**	**BV**
Grøntved et al. (355)	435	DNK	Adolescence->adulthood	ST >3 h/day	3.46	•		•		
Xie et al. (284)	2,134	CHN	University students	Worst quartile	3.20	•		•		
Zhang et al. (350)	1,196	GER	Women 18–25	PA < 1/mon.	2.08	•				
Wu et al. (307)	2,521	CHN	University students	ST increase	1.77	•		•		
Sui et al. (356)	4,802	USA	Patients 18–80	Worst tertile	1.74	•		•		
Pavey and Brown (357)	6,205	AUS	Adult women	>10 h/day	1.72	•		•		
Tsutsumimoto et al. (351)	3,066	JPN	Older adults 65+	>8 h/day	1.64	•		•		
Cabello et al. (352)	7,908	Int.	Adults 18–49 and 50+	low PA	1.23	•		•		
Zink et al. (358)	2,525	USA	High school students	>4 h/day	1.23			•		
Vancampfort et al. (296)	5,483	IRL	Adults 50+	>8 h/day	1.06	•		•		
**Diet quality (*****N*** = **1)**						**TO**	**CA**	**MI**	**TR**	**BV**
Knüppel et al. (342)	8,087	UK	Civil servants 35–55	Worst tertile	1.47	•		•		
	**Median**	**Unweighted mean (SD)**		**Weighted mean**						
Cannabis	1.07	1.23 (0.42)		1.14						
Tobacco	1.47	1.53 (0.36)		1.40						
Sedentary behavior	1.73	1.91 (0.81)		1.60						
Diet quality	1.47	1.47		1.47						
**Cannabis (*****N*** = **6)**	***N***	**Area**	**Sample**	**Outcome**	**OR**	**SD**	**TO**	**MI**	**TR**	**BV**
Bolanis et al. (359)	1,606	CAN	Adolescents	Weekly	2.30		•			
Davies et al. (171)	2,602	UK	Adolescence->adulthood	Problem	2.00			•		
Feingold et al. (348)	34,653	USA	Adults 18+	Any use	1.72		•	•		
Cougle et al. (213)	34,653	USA	Adults	Dependence	1.33			•		
Bierhoff et al. (360)	2,397	USA	University students	Past 30 days	1.02			•		
Stapinski et al. (361)	1,602	CHL	Adolescents	Any use	0.98		•	•		
**Tobacco (*****N*** = **5)**						**SD**	**CA**	**MI**	**TR**	**BV**
Wiernik et al. (257)	30,818	FRA	Adults 18–69	Curr. E-cig	2.02			•		
Davies et al. (171)	2,627	UK	Adolescence->adulthood	Weekly +	1.23			•		
Bierhoff et al. (360)	2,397	USA	University students	Past 30 days	1.05			•		
Lechner et al. (362)	2,460	USA	Adolescents	Any	1.02			•		
Cougle et al. (213)	34,653	USA	Adults	Dependence	0.80			•		
**Sedentary behavior (*****N*** = **1)**						**TO**	**CA**	**MI**	**TR**	**BV**
Zink et al. (358)	2,525	USA	High school students	>4 h/day	1.36			•		
**Diet quality (*****N*** = **1)**						**TO**	**CA**	**MI**	**TR**	**BV**
Knüppel et al. (342)	8,087	UK	Civil servants 35–55	Increase	0.98	•		•		
	**Median**	**Unweighted mean (SD)**		**Weighted mean**						
Cannabis	1.53	1.56 (0.54)		1.53						
Tobacco	1.05	1.22 (0.47)		1.35						
Sedentary behavior	1.36	1.36		1.36						
Diet quality	0.98	0.98		0.98						

OR, odds ratio; SD, sedentary behavior and diet quality; TO, tobacco; CA, cannabis; MI, social misery; TR, psychological trauma; BV, bivariate (unadjusted); PA, physical activity; SB, sedentary behavior; ST, screen time.

#### Anxiety

The review included 93 cross-sectional and 19 longitudinal figures for the association between unhealthy behaviors and anxiety (Tables 3a, 3b). The cross-sectional studies provide convincing evidence for associations between anxiety and cannabis, tobacco, sedentary behavior, and diet quality. The longitudinal studies provide some evidence that sedentary behavior and use of cannabis and tobacco may lead to subsequent anxiety and tenuous evidence that anxiety may lead to subsequent sedentary behavior and cannabis and tobacco use. No longitudinal evidence related to diet quality was included in this review.

**Table 3a:** Cross-sectional anxiety studies (*N* = 93).

	***N***	**Area**	**Sample**	**Exposure**	**OR**	**Covariate adjustments**					
**Cannabis (*****N*** = **23)**						**SD**	**TO**	**MI**	**TR**	**BV**
Hasin et al. (136)	36,309	USA	Adults 18+	Disorder	3.70			•		
Hasin and Walsh (20)	36,309	USA	Adults 18+	Lifetime disorder	3.20			•		
Padwa et al. (151)	8,940	USA	Adult patients and contr.	Any	2.74		•			
Keith et al. (216)	1,776	USA	University students	Weekly 1–2	2.30					
Cougle et al. (211)	43,093	USA	Adults	Weekly	2.20			•		
Hines et al. (182)	1,087	UK	Adults	High-potency	1.92			•		
Young-Wolff et al. (123)	196,022	USA	Pregnant women	During pregnancy	1.90			•		
Cougle et al. (213)	43,093	USA	Adults	Weekly	1.89			•		
Hill et al. (215)	3,157	USA	Military veterans	Non-CUD	1.52			•		
López-Gil et al. (363)	14,516	ARG	Adolescents	Monthly	1.46	•	•	•		
Mannes et al. (217)	932	USA	Adult patients	Past year	1.28			•		
Kerridge et al. (224)	36,309	USA	Adults 18+	Disorder	1.25		•	•		
Hill et al. (229)	4,069	USA	Military veterans	Past 6 months	1.23			•	•	
Butler et al. (220)	6,550	CAN	Adolescents	Any	1.20		•	•		
Campbell et al. (176)	101,405	USA	Patients 18+	Disorder	1.16		•	•		
Prestage et al. (223)	3,017	AUS	Gay and bisexual men	Past 6 months	1.16					ø
Lekoubou et al. (230)	400,391	USA	Patients 18+	Disorder	1.14			•		
Welsh et al. (234)	483	USA	Patients 11–24	Disorder	1.12					
Gaete et al. (236)	935	CHL	Young offenders 14–23	Any	1.09		•	•	•	
Chan et al. (183)	181,870	Int.	Adults 18+	Potent herb	1.05			•		
Bonsaksen et al. (231)	4,527	NOR	Adults 18+	Any	0.96			•		
Thompson et al. (233)	662	CAN	Adolescence->adulthood	>1/week	0.94		•	•		
Risal et al. (208)	2,100	NPL	Adults 18–65	Any	0.90					ø
**Tobacco (*****N*** = **28)**						**SD**	**CA**	**MI**	**TR**	**BV**
Klein et al. (239)	11,785	USA	Children 9–12	Any	2.97			•		
Hajure et al. (364)	411	ETH	Adult patients	Current	2.27			•		
Nakie et al. (365)	810	ETH	High school students	Mod. risk	2.03			•		
Cougle et al. (213)	43,093	USA	Adults	Daily	1.99			•		
Peltzer and Pengpid (366)	11,124	IDN	Adolescents	Past 30 days	1.95	•		•	•	
Sumbe et al. (273)	2,439	USA	Adolescents and adults	Past 30 days	1.90		•	•		
López-Sánchez et al. (133)	4,157	ESP	Adult patients	Current	1.76	•		•		
Prestage et al. (223)	3,017	AUS	Gay and bisexual men	Daily	1.73		•	•		
Mannes et al. (217)	932	USA	Adult patients	Past 30 days	1.71			•		
Kelishadi et al. (242)	13,486	IRN	Children 6–18	Daily	1.70			•		
Hruby et al. (281)	12,708	USA	Military service members	Past 30 days	1.70	•	•	•		
Cougle et al. (211)	43,093	USA	Adults	Weekly	1.69			•		
Islam et al. (252)	563	BGD	Adolescents	Current	1.67	•		•		
López-Gil et al. (363)	16,872	ARG	Adolescents	Monthly	1.59	•	•	•		
Matcham et al. (253)	7,878	UK	Adult patients	Current	1.44					
Bourbon et al. (125)	10,985	FRA	University students	Daily	1.40		•	•	•	
**Tobacco (*****N*** = **28)**						**SD**	**CA**	**MI**	**TR**	**BV**
Kelishadi et al. (242)	13,486	IRN	Children 6–18	Secondhand	1.28			•		
Kastaun et al. (274)	11,937	GER	Adults 18+	Current	1.22			•		
Parker et al. (367)	36,309	USA	Adults	Past year	1.20		•	•		
Ellis et al. (275)	3,468	USA	Adults	Any	1.18			•		
Chou et al. (271)	36,309	USA	Adults 18+	Disorder	1.15			•		
Abbasi-Ghahramanloo et al. (368)	2,434	IRN	Male workers	Current	1.13		•	•		
Li et al. (278)	1,504	CHN	University students	Current	1.12					ø
Asfaw et al. (369)	523	ETH	University students	Current	1.10					
Shahwan et al. (134)	6,126	SGP	Adults 18+	Dependence	1.00					
Chou et al. (280)	36,309	USA	Adults 18+	Lifetime e-cig.	1.00		•	•		
Risal et al. (208)	2,100	NPL	Adults 18–65	Any	0.60					ø
Welsh et al. (234)	483	USA	Patients 11–24	Disorder	0.28					
**Sedentary behavior (*****N*** = **29)**						**TO**	**CA**	**MI**	**TR**	**BV**
Chen et al. (370)	1,331	CHN	Adolescents	Games >6 h/day	5.25			•		
Humer et al. (283)	6,703	AUT	Adolescents	Phone >8 h/day	3.96					
Jiang et al. (289)	28,298	CHN	University students	Sedentary	3.57	•		•		
Wen et al. (371)	900	CHN	High school students	Scr. T. >2 h/day	2.56				•	
Vancampfort et al. (372)	181,093	Int.	Adolescents	>8 h/day	2.27			•		
Bu et al. (373)	1,846	CHN	University students	Low pa	2.20			•		
Kim et al. (196)	6,510	KOR	Adults 18–64	Addiction	2.19			•		
Vadlin et al. (290)	2,110	SWE	Patients and controls	Problematic gaming	2.06				•	
Vancampfort et al. (374)	42,469	Int.	Adults 18+	>8 h/day	2.04	•		•		
Kang et al. (375)	1,204	KOR	Older adults 65+	Low PA	1.77			•		
Liu et al. (300)	13,659	CHN	Adolescents	Games >2 h/day	1.59	•		•		
Draper et al. (320)	1,719	ZAF	Women 18–26	Low PA	1.54					
Wu et al. (297)	4,747	CHN	University students	Scr. t. >2 h/day	1.49			•		
Lu et al. (306)	965	CHN	Adolescents	>4 h/day	1.47			•		
Vancampfort et al. (293)	4,082	Int.	Adult patients	>8 h/day	1.41	•		•		
Zhang et al. (303)	27,723	CHN	University students	scr. t. >4 h/day	1.39					
López-Gil et al. (363)	31,388	ARG	Adolescents	>3 h/day	1.33	•	•	•		
Islam et al. (252)	563	BGD	Adolescents	Low PA	1.31					ø
Taheri et al. (319)	13,486	IRN	Children 6–18	scr. t. >2 h/day	1.28	•		•		
López-Sánchez et al. (133)	4,157	ESP	Adult patients	Inactivity	1.26	•		•		
Wu et al. (307)	2,521	CHN	University students	scr. t. >2 h/day	1.25	•		•		
Wang et al. (376)	59,587	Int.	Adolescents	>2 h/day	1.22	•			•	
Hruby et al. (281)	12,708	USA	Military service members	PA < 75 min/week	1.21	•	•	•		
Padmapriya et al. (321)	863	SGP	Pregnant women 18+	TV >2 h/day	1.21	•		•		
Pengpid and Peltzer (318)	3,201	Int.	Adult patients	>8 h/day	1.17	•		•		
Schuch et al. (311)	937	BRA	Adults 18+	>10 h/day	1.17	•		•		
Werneck et al. (377)	99,791	BRA	Adolescents	TV >4 h/day	1.16			•		
McDowell et al. (69)	3,165	IRL	Adults 50+	Low PA	1.06	•		•		
Song and Lee (378)	53,510	KOR	Adolescents	Low PA	1.06	•	•		•	
**Diet quality (*****N*** = **13)**						**TO**	**CA**	**MI**	**TR**	**BV**
ElBarazi and Tikamd (322)	509	EGY	University students	Daily junk food	11.10					ø
Khan and Uddin (379)	2,742	BGD	Adolescents	Fast fo. 3+ day/week	2.64	•		•		
Hall et al. (343)	444	MEX	University students	Worst half	2.35	•				
Zhang et al. (303)	27,723	CHN	University students	Soda >4/day	2.06					
Werneck et al. (380)	100,648	BRA	Adolescents	Daily fast food	1.96					
Mutiso et al. (198)	9,742	KEN	High school students	Binge eating	1.68	•	•	•		
Kaufman-Shriqui et al. (381)	3,797	Int.	Adults 18+	Worsening diet	1.61	•		•		
Werneck et al. (377)	99,791	BRA	Adolescents	Daily poor	1.47			•		
López-Gil et al. (363)	16,872	ARG	Adolescents	Fast food 1/week	1.24	•	•	•		
Sangsefidi et al. (331)	9,965	IRN	Adults 20–70	Fast food 1/week	1.19	•		•		
Liu et al. (325)	1,311	CHN	Children 7–17	Daily soda	1.10			•		
Sze et al. (152)	424	CHN	University students	Emotional eating	0.99			•		
Hosseinzadeh et al. (344)	3,846	IRN	Adults 20–55	Worst quintile	0.86	•		•		
	**Median**	**Unweighted mean (SD)**		**Weighted mean**						
Cannabis	1.25	1.62 (0.76)		1.50						
Tobacco	1.52	1.49 (0.53)		1.51						
Sedentary behavior	1.41	1.81 (0.97)		1.76						
Diet quality	1.61	2.33 (2.69)		1.71						

OR, odds ratio; SD, sedentary behavior and diet quality; TO, tobacco; CA, cannabis; MI, social misery; TR, psychological trauma; BV, bivariate (unadjusted); PA, physical activity.

**Table 3b:** Longitudinal anxiety studies (*N* = 19).

**Longitudinal: Behavior before disorder (*****N*** = **11)**					**Covariate adjustments**					
**Cannabis (*****N*** = **6)**	***N***	**Area**	**Sample**	**Exposure**	**OR**	**SD**	**TO**	**MI**	**TR**	**BV**
Green et al. (382)	330	USA	Urban blacks	Before age 16	2.12		•	•	•	
Feingold et al. (383)	34,653	USA	Adults	Weekly +	1.50		•	•		
Copeland et al. (141)	1,420	USA	Childhood->adulthood	Cumul. daily	1.20		•	•	•	
Hengartner et al. (345)	591	SWI	Adolescence->adulthood	Adolescence	1.12			•		
Cougle et al. (211)	34,653	USA	Adults	Weekly	1.09			•		
Blanco et al. (347)	34,653	USA	Adults 18+	Past year	1.00		•	•	•	
**Tobacco (*****N*** = **2)**						**SD**	**CA**	**MI**	**TR**	**BV**
Rognli et al. (204)	2,602	NOR	Adolescence->adulthood	Low dep.	1.96			•		
Cougle et al. (211)	34,653	USA	Adults	Weekly	1.40			•		
**Sedentary behavior (*****N*** = **3)**					**TO**	**CA**	**MI**	**TR**	**BV**	
Wu et al. (307)	2,521	CHN	University students	ST increase	1.98	•		•		
Zink et al. (358)	2,525	USA	High school students	>4 h/day	1.54			•		
McDowell et al. (69)	3,165	IRL	Adults 50+	Low PA	1.06	•		•		
	**Median**	**Unweighted mean (SD)**		**Weighted mean**						
Cannabis	1.16	1.34 (0.42)		1.20						
Tobacco	1.68	1.68 (0.40)		1.44						
Sedentary behavior	1.54	1.53 (0.46)		1.49						
**Longitudinal: disorder before behavior (*****N*** = **8)**					**Covariate adjustments**					
**Cannabis (*****N*** = **5)**	***N***	**Area**	**Sample**	**Outcome**	**OR**	**SD**	**TO**	**MI**	**TR**	**BV**
Tran et al. (384)	1,230	USA	Military veterans	Past 30 days	6.53			•		
Stapinski et al. (361)	1,602	CHL	Adolescents	Any use	1.25		•	•		
Bierhoff et al. (360)	2,397	USA	University students	Past 30 days	1.01			•		
Cougle et al. (213)	34,653	USA	Adults	Dependence	0.75			•		
Feingold et al. (383)	34,653	USA	Adults	Any use	0.49		•	•		
**Tobacco (*****N*** = **2)**						**SD**	**CA**	**MI**	**TR**	**BV**
Cougle et al. (213)	34,653	USA	Adults	Dependence	1.17			•		
Bierhoff et al. (360)	2,397	USA	University students	Past 30 days	1.02			•		
**Sedentary behavior (*****N*** = **1)**						**TO**	**CA**	**MI**	**TR**	**BV**
Zink et al. (358)	2,525	USA	High school students	Gaming >4 h/day	1.36			•		
	**Median**	**Unweighted mean (SD)**		**Weighted mean**						
Cannabis	1.01	2.01 (2.54)		0.74						
Tobacco	1.10	1.10 (0.11)		1.16						
Sedentary behavior	1.36	1.36		1.36						

OR, odds ratio; SD, sedentary behavior and diet quality; TO, tobacco; CA, cannabis; MI, social misery; TR, psychological trauma; BV, bivariate (unadjusted); PA, physical activity.

#### Bipolar disorder

This review confirms that there is limited research on the association between unhealthy behaviors and bipolar disorder. A total of 25 cross-sectional and 10 longitudinal figures were included in the review (Tables 4a, 4b), many of which were based on the same NESARC dataset. The cross-sectional studies provide convincing evidence for associations between bipolar disorder and cannabis and tobacco use and tenuous evidence for associations with sedentary behavior and diet quality. The longitudinal studies provide some evidence that the use of cannabis and tobacco may lead to subsequent bipolar disorder, but no evidence that bipolar disorder may lead to subsequent cannabis and tobacco use. No longitudinal evidence related to sedentary behavior and diet quality was included in this review.

**Table 4a:** Cross-sectional bipolar studies (*N* = 25).

	***N***	**Area**	**Sample**	**Exposure**	**OR**	**Covariate adjustments**					
**Cannabis (*****N*** = **9)**						**SD**	**TO**	**MI**	**TR**	**BV**
Hasin et al. (136)	36,309	USA	Adults 18+	Disorder	3.85			•		
Hasin and Walsh (20)	36,309	USA	Adults 18+	Lifetime disorder	3.30			•		
Cougle et al. (211)	43,093	USA	Adults	Weekly	2.62			•		
Padwa et al. (151)	8,940	USA	Adult patients and contr.	Any	2.54		•			
Patel et al. (385)	380,265	USA	Adult patients	Disorder	1.53		•			
Lekoubou et al. (230)	400,391	USA	Patients 18+	Disorder	1.45			•		
Kerridge et al. (224)	36,309	USA	Adults 18+	Disorder	1.42		•	•		
Cougle et al. (213)	43,093	USA	Adults	Weekly	1.27			•		
Campbell et al. (176)	101,405	USA	Patients 18+	Disorder	1.16		•	•		
**Tobacco (*****N*** = **13)**						**SD**	**CA**	**MI**	**TR**	**BV**
Shahwan et al. (134)	6,126	SGP	Adults 18+	Dependence	3.70					
Molla et al. (186)	422	ETH	Adult patients	Dependence	2.76			•		
Li et al. (51)	1,102	CHN	Adult patients	Daily	2.50			•		
Dickerson et al. (187)	1,938	USA	Adult patients and contr.	Current	2.18			•		
Cougle et al. (213)	43,093	USA	Adults	Daily	2.10			•		
Cougle et al. (211)	43,093	USA	Adults	Weekly	1.82			•		
Yeh et al. (386)	2,799	TWN	Adolescents	Regular	1.59		•			
Bourbon et al. (125)	10,985	FRA	University students	Daily	1.50		•	•	•	
Chou et al. (271)	36,309	USA	Adults 18+	Disorder	1.49			•		
Chou et al. (280)	36,309	USA	Adults 18+	Lifetime e-cig.	1.30		•	•		
Patel et al. (385)	380,265	USA	Adult patients	Disorder	0.92		•			
Werneck et al. (189)	60,202	BRA	Adult patients and contr.	Current	0.89			•		
Zolezzi et al. (188)	196	QAT	Patients 15+	Current	0.54					ø
**Sedentary behavior (*****N*** = **2)**						**TO**	**CA**	**MI**	**TR**	**BV**
Kim et al. (196)	6,510	KOR	Adults 18–64	Addiction	3.12			•		
Werneck et al. (189)	60,202	BRA	Adult patients and contr.	TV >5 h/day	0.41			•		
**Diet quality (*****N*** = **1)**						**TO**	**CA**	**MI**	**TR**	**BV**
Werneck et al. (189)	60,202	BRA	Adult patients and contr.	Daily sweets	2.20			•		
	**Median**	**Unweighted mean (SD)**		**Weighted mean**						
Cannabis	1.53	2.13 (0.98)		1.64						
Tobacco	1.59	1.79 (0.86)		1.16						
Sedentary behavior	1.77	1.77 (1.92)		0.67						
Diet quality	2.20	2.20		2.20						

OR, odds ratio; SD, sedentary behavior and diet quality; TO, tobacco; CA, cannabis; MI, social misery; TR, psychological trauma; BV, bivariate (unadjusted).

**Table 4b:** Longitudinal bipolar studies (*N* = 10).

**Longitudinal: behavior before disorder (*****N*** = **7)**					**Covariate adjustments**					
**Cannabis (*****N*** = **4)**	***N***	**Area**	**Sample**	**Exposure**	**OR**	**SD**	**TO**	**MI**	**TR**	**BV**
Feingold et al. (348)	34,653	USA	Adults 18+	Weekly +	2.47		•	•		
Marwaha et al. (387)	3,370	UK	Adolescence->adulthood	Weekly 2+	2.21			•	•	
Cougle et al. (211)	34,653	USA	Adults	Weekly	1.37			•		
Blanco et al. (347)	34,653	USA	Adults 18+	Past year	1.30		•	•	•	
**Tobacco (*****N*** = **3)**						**SD**	**CA**	**MI**	**TR**	**BV**
Rognli et al. (204)	2,602	NOR	Adolescence->adulthood	Daily, low dep.	4.21			•		
Bolstad et al. (354)	7,660	FIN	Adolescence->adulthood	Daily	1.83		•	•		
Cougle et al. (211)	34,653	USA	Adults	Weekly	1.53			•		
	**Median**	**Unweighted mean (SD)**		**Weighted mean**						
Cannabis	1.79	1.84 (0.59)		1.73						
Tobacco	1.83	2.52 (1.47)		1.74						
**Longitudinal: disorder before behavior (*****N*** = **3)**					**Covariate adjustments**					
**Cannabis (*****N*** = **2)**	***N***	**Area**	**Sample**	**Outcome**	**OR**	**SD**	**TO**	**MI**	**TR**	**BV**
Feingold et al. (348)	34,653	USA	Adults 18+	Any use	0.61		•	•		
Cougle et al. (213)	34,653	USA	Adults	Dependence	0.45			•		
**Tobacco (*****N*** = **1)**						**SD**	**CA**	**MI**	**TR**	**BV**
Cougle et al. (213)	34,653	USA	Adults	Dependence	1.14			•		
	**Median**	**Unweighted mean (SD)**		**Weighted mean**						
Cannabis	0.53	0.53 (0.11)		0.53						
Tobacco	1.14	1.14		1.14						

OR, odds ratio; SD, sedentary behavior and diet quality; TO, tobacco; CA, cannabis; MI, social misery; TR, psychological trauma; BV, bivariate (unadjusted).

#### Personality disorders

The findings of this review confirm that the association between unhealthy behaviors and personality disorders has not been intensively studied. A total of 16 cross-sectional and three longitudinal figures were included in the review (Tables 5a, 5b), many of which were based on the same NESARC dataset. The cross-sectional studies provide convincing evidence for associations between personality disorder and cannabis and tobacco use, but no evidence related to sedentary behavior and diet quality was included in this review. Furthermore, no evidence indicating that unhealthy behaviors may lead to subsequent personality disorder and no evidence that personality disorder may lead to subsequent unhealthy behavior was included in this review.

**Table 5a:** Cross-sectional personality disorder studies (*N* = 16).

	***N***	**Area**	**Sample**	**Exposure**	**OR**	**Covariate adjustments**					
**Cannabis (*****N*** = **9)**						**SD**	**TO**	**MI**	**TR**	**BV**
Hasin et al. (136)	36,309	USA	Adults 18+	Disorder	4.80			•		
Hasin and Walsh (20)	36,309	USA	Adults 18+	Lifetime disorder	4.70			•		
Jemal et al. (388)	401	ETH	Adult patients	Current	4.38			•		
Estévez et al. (221)	5,677	SWI	Male military conscripts	Weekly >1	4.00			•		
Cougle et al. (213)	43,093	USA	Adults	Weekly	3.37			•		
Cougle et al. (211)	43,093	USA	Adults	Weekly	3.27			•		
Holzer et al. (389)	31,765	USA	Adults 50+	Disorder	2.94			•		
Kerridge et al. (224)	36,309	USA	Adults 18+	Disorder	2.55		•	•		
Fink et al. (232)	392	USA	Adult patients	Mod. disorder	1.35			•		
**Tobacco (*****N*** = **7)**						**SD**	**CA**	**MI**	**TR**	**BV**
Goldstein et al. (390)	36,309	USA	Adults 18+	Disorder	2.70		•	•		
Estévez et al. (221)	5,677	SWI	Men 19	Daily	2.57			•		
Holzer et al. (389)	31,765	USA	Adults 50+	Disorder	2.31			•		
Cougle et al. (213)	43,093	USA	Adults	Daily	1.93			•		
Chou et al. (280)	36,309	USA	Adults 18+	Lifetime e-cig.	1.70		•	•		
Cougle et al. (211)	43,093	USA	Adults	Weekly	1.69			•		
Chou et al. (271)	36,309	USA	Adults 18+	Disorder	1.55			•		
	**Median**	**Unweighted mean (SD)**		**Weighted mean**						
Cannabis	3.37	3.48 (1.12)		3.61						
Tobacco	1.93	2.06 (0.46)		1.98						

OR, odds ratio; SD, sedentary behavior and diet quality; TO, tobacco; CA, cannabis; MI, social misery; TR, psychological trauma; BV, bivariate (unadjusted).

**Table 5b:** Longitudinal personality disorder studies (*N* = 3).

**Longitudinal: behavior before disorder (*****N*** = **1)**					**Covariate adjustments**					
**Cannabis (*****N*** = **1)**	***N***	**Area**	**Sample**	**Exposure**	**OR**	**SD**	**TO**	**MI**	**TR**	**BV**
Carpenter et al. (391)	34,481	USA	Adults 18+	Disorder	1.27			•		
	**Median**	**Unweighted mean**		**Weighted mean**						
Cannabis	1.27	1.27		1.27						
**Longitudinal: disorder before behavior (*****N*** = **2)**					**Covariate adjustments**					
**Cannabis (*****N*** = **1)**	***N***	**Area**	**Sample**	**Outcome**	**OR**	**SD**	**TO**	**MI**	**TR**	**BV**
Cougle et al. (213)	34,653	USA	Adults	Dependence	1.00			•		
**Tobacco (*****N*** = **1)**						**SD**	**CA**	**MI**	**TR**	**BV**
Cougle et al. (213)	34,653	USA	Adults	Dependence	1.05			•		
	**Median**	**Unweighted mean**		**Weighted mean**						
Cannabis	1.00	1.00		1.00						
Tobacco	1.05	1.05		1.05						

OR, odds ratio; SD, sedentary behavior and diet quality; TO, tobacco; CA, cannabis; MI, social misery; TR, psychological trauma; BV, bivariate (unadjusted).

#### Attention-deficit/hyperactivity disorder

This review included 36 cross-sectional and 19 longitudinal figures for the association between unhealthy behaviors and ADHD (Tables 6a, 6b). The cross-sectional studies provide convincing evidence for associations between ADHD and cannabis use, tobacco use, sedentary behavior, and diet quality. The longitudinal studies focused for the most part on how the disorder may lead to unhealthy behavior, probably because ADHD is a disorder often diagnosed in childhood. As such, only single studies provide any evidence that sedentary behavior and poor diet quality may lead to subsequent ADHD, and no evidence at all was included for cannabis and tobacco use. However, there was some evidence that ADHD may lead to subsequent cannabis and tobacco use and single-study evidence that the disorder may lead to sedentary behavior and poor diet quality.

**Table 6a:** Cross-sectional ADHD studies (*N* = 36).

	***N***	**Area**	**Sample**	**Exposure**	**OR**	**Covariate adjustments**					
**Cannabis (*****N*** = **8)**						**SD**	**TO**	**MI**	**TR**	**BV**
Capusan et al. (392)	17,779	SWE	Adult twins	Any	2.19			•		
Welsh et al. (234)	483	USA	Patients 11–24	Disorder	2.10					
Karlsson et al. (393)	4,666	SWE	Adolescents	Any	1.91			•		
Estévez et al. (221)	5,677	SWI	Male military conscripts	Weekly >1	1.84			•		
Moulin et al. (394)	1,214	FRA	Adults 18–35	Abuse	1.80		•	•	•	
Fuller-Thom et al. (395)	6,872	CAN	Adults 20–39	Disorder	1.46		•	•	•	
Thompson et al. (233)	662	CAN	Adolescence->adulthood	>1/week	1.35		•	•		
Leventhal et al. (222)	3,177	USA	Adolescents	Past-30 days	1.27		•	•		
**Tobacco (*****N*** = **11)**						**SD**	**CA**	**MI**	**TR**	**BV**
Riegler et al. (396)	3,280	AUT	Male military conscripts	Abuse	3.01					ø
Moulin et al. (394)	1,214	FRA	Adults 18–35	>10 cig./day	2.00			•	•	
Yeh et al. (386)	2,799	TWN	Adolescents	Regular	1.69		•			
Estévez et al. (221)	5,677	SWI	Men 19	Daily	1.62			•		
Yeom et al. (397)	3,441	KOR	Male military conscripts	Dependence	1.61			•		
Galéra et al. (398)	8,110	FRA	University students	Daily	1.52			•		
Xu et al. (399)	195,443	USA	University students	Daily	1.49	•				
Capusan et al. (392)	18,167	SWE	Adult twins	Daily	1.33			•		
Welsh et al. (234)	483	USA	Patients 11–24	Disorder	0.89					
Weissenberg et al. (400)	1,012	CZE	Adults 18–65	Current	0.68	•	•			
Abbasi-Ghahramanloo et al. (368)	2,434	IRN	Male workers	Current	0.61		•	•		
**Sedentary behavior (*****N*** = **10)**						**TO**	**CA**	**MI**	**TR**	**BV**
Kim et al. (196)	6,510	KOR	Adults 18–64	Addiction	8.90			•		
Chowdhury (401)	310	BGD	Adults 18+	Phone >4 h/day	7.27			•		
Gul et al. (402)	289	TUR	Adolescent patients	FB overuse	5.48					ø
Alageel et al. (291)	506	Int.	University students	Addiction	2.71	•				
Vadlin et al. (290)	2,110	SWE	Patients and controls	Problematic gaming	2.43				•	
Liu et al. (300)	13,659	CHN	Adolescents	Games >2 h/day	2.35	•		•		
Moulin et al. (394)	1,214	FRA	Adults 18–35	Games >1/week	1.40		•	•	•	
Zhang et al. (197)	7,121	CHN	Adults 18–81	TV >3 h/day	1.16	•		•		
Claesdotter-Knutsson et al. (403)	17,006	SWE	Adolescents	High phone	1.14	•	•			
Cook et al. (404)	45,897	USA	Youth 10–17	SB >2 h/day	1.05			•		
**Diet quality (*****N*** = **7)**						**TO**	**CA**	**MI**	**TR**	**BV**
Ríos-Hernán et al. (405)	120	ESP	Child patients and contr.	Soda (w. tert.)	3.89					ø
Yu et al. (406)	332	TWN	Child patients and contr.	Daily soda	3.69			•		
Abbasi et al. (407)	500	IRN	Child patients and contr.	Worst quintile	3.45			•		
Yan et al. (408)	14,912	CHN	Preschoolers 3–6	Worst quintile	1.76			•		
Kim et al. (409)	16,831	KOR	Children 6–12	Daily soda	1.75			•		
Weissenberg et al. (400)	1,012	CZE	Adults 18–65	Daily sweets	1.37	•	•			
Zhou et al. (410)	592	CHN	Child patients and contr.	Worst tertile	1.25			•	•	
	**Median**	**Unweighted mean (SD)**		**Weighted mean**						
Cannabis	1.82	1.74 (0.34)		1.89						
Tobacco	1.52	1.50 (0.67)		1.50						
Sedentary behavior	2.39	3.39 (2.82)		1.88						
Diet quality	1.76	2.45 (1.17)		1.79						

OR, odds ratio; SD, sedentary behavior and diet quality; TO, tobacco; CA, cannabis; MI, social misery; TR, psychological trauma; BV, bivariate (unadjusted); PA, physical activity; SB, sedentary behavior.

**Table 6b:** Longitudinal ADHD studies (*N* = 19).

**Longitudinal: behavior before disorder (*****N*** = **2)**					**Covariate adjustments**					
**Sedentary behavior (*****N*** = **1)**	***N***	**Area**	**Sample**	**Exposure**	**OR**	**TO**	**CA**	**MI**	**TR**	**BV**
Poulain et al. (411)	527	GER	Preschool patients 3–6	ST >0.5 h/day	3.36			•		
**Diet quality (*****N*** = **1)**						**TO**	**CA**	**MI**	**TR**	**BV**
Del-Ponte et al. (412)	2,924	BRA	Child patients	Worst tertile	1.67			•		
	**Median**	**Unweighted mean**		**Weighted mean**				
Sedentary behavior	3.36	3.36		3.36				
Diet quality	1.67	1.67		1.67				
**Longitudinal: disorder before behavior (*****N*** = **17)**					**Covariate adjustments**					
**Cannabis (*****N*** = **6)**	***N***	**Area**	**Sample**	**Outcome**	**OR**	**SD**	**TO**	**MI**	**TR**	**BV**
Jean et al. (413)	4,270	FRA	University students	Monthly >1	1.86			•	•	
Vogel et al. (414)	5,103	SWI	Male military conscripts	Past year	1.85			•		
Kim and Kim (415)	2,449	USA	Siblings	Any use	1.77					
Moggi et al. (416)	4,602	SWI	Male military conscripts	Past year	1.70			•		
Bierhoff et al. (360)	2,397	USA	University students	Past 30 days	1.11			•		
Estévez-Lamorte et al. (417)	4,975	SWI	Male military conscripts	Weekly >1	0.75			•		
**Tobacco (*****N*** = **9)**						**SD**	**CA**	**MI**	**TR**	**BV**
Kim and Kim (415)	2,449	USA	Siblings	Regular	3.45					
Mitchell et al. (418)	709	CAN	Patients and controls	Daily	2.59			•		
Jaisoorya et al. (116)	5,145	IND	University students	Any use	1.96					
Moggi et al. (416)	4,602	SWI	Male military conscripts	Past year	1.62			•		
Elkins et al. (419)	3,762	USA	Twin pairs 11 and 17	Daily	1.29			•		
Estévez-Lamorte et al. (417)	4,968	SWI	Male military conscripts	Daily	1.16			•		
Vogel et al. (414)	5,103	SWI	Male military conscripts	Past year	1.07			•		
Bierhoff et al. (360)	2,397	USA	University students	Past 30 days	1.04			•		
Goldenson et al. (420)	1,921	USA	Adolescents	Any use	1.03			•		
**Sedentary behavior (*****N*** = **1)**						**TO**	**CA**	**MI**	**TR**	**BV**
Bowling et al. (421)	8,250	USA	Children 6–15	PA < 3 day/week	1.17			•		
**Diet quality (*****N*** = **1)**						**TO**	**CA**	**MI**	**TR**	**BV**
Bowling et al. (421)	8,250	USA	Children 6–15	Poor diet	1.46			•		
	**Median**	**Unweighted mean (SD)**		**Weighted mean**						
Cannabis	1.74	1.51 (0.47)		1.51						
Tobacco	1.29	1.69 (0.84)		1.56						
Sedentary behavior	1.17	1.17		1.17						
Diet quality	1.46	1.46		1.46						

OR, odds ratio; SD, sedentary behavior and diet quality; TO, tobacco; CA, cannabis; MI, social misery; TR, psychological trauma; BV, bivariate (unadjusted); PA, physical activity; ST, screen time.

#### Post-traumatic stress disorder

The review included 35 cross-sectional and only two longitudinal figures for the association between unhealthy behaviors and PTSD (Tables 7a, 7b). The cross-sectional studies provide convincing evidence for associations between PTSD and cannabis and tobacco use, as well as some evidence for an association with sedentary behavior, but none for diet quality. The longitudinal studies provide single-study evidence that the use of cannabis and tobacco may lead to subsequent PTSD. Nevertheless, it should be noted that sensitivity analyses for specialty samples, behavioral measurement disparities, and control variables introduced discrepancies related to the cannabis-PTSD relationship, indicating that these findings should be interpreted with extra carefulness.

**Table 7a:** Cross-sectional PTSD studies (*N* = 35).

	***N***	**Area**	**Sample**	**Exposure**	**OR**	**Covariate adjustments**					
**Cannabis (*****N*** = **18)**						**SD**	**TO**	**MI**	**TR**	**BV**
Metrik et al. (205)	301	USA	Male military veterans	Weekly 2+	5.96					
Hasin et al. (136)	36,309	USA	Adults 18+	Disorder	4.30			•		
de Silva et al. (422)	671	LKA	Male military personnel	Past year	4.20			•		
Hasin and Walsh (20)	36,309	USA	Adults 18+	Lifetime disorder	3.80			•		
Padwa et al. (151)	8,940	USA	Adult patients and contr.	Any	3.41		•			
Young-Wolff et al. (123)	196,022	USA	Pregnant women	During pregnancy	2.82			•		
Ehlers et al. (423)	614	USA	Mexican Am. 18–30	Dependence	2.02					ø
Hill et al. (215)	3,157	USA	Military veterans	Non-CUD	1.96			•		
Bilevicius et al. (424)	36,309	USA	Adults 18+	Disorder	1.94		•	•		
Kerridge et al. (224)	36,309	USA	Adults 18+	Disorder	1.65		•	•		
Alenko et al. (425)	398	ETH	Male drivers	Moderate risk	1.55		•	•		
Welsh et al. (234)	483	USA	Patients 11–24	Disorder	1.52					
Gentes et al. (426)	719	USA	Male military veterans	Past 6 months	1.30		•	•		
Kevorkian et al. (427)	2,990	USA	Adults 18+	Disorder	1.22					
Lekoubou et al. (230)	400,391	USA	Patients 18+	Disorder	1.13			•		
Campbell et al. (176)	101,405	USA	Patients 18+	Disorder	1.00		•	•		
Hill et al. (229)	4,069	USA	Military veterans	Past 6 months	1.00			•	•	
Fink et al. (232)	392	USA	Adult patients	Mod. disorder	0.57			•		
**Tobacco (*****N*** = **12)**						**SD**	**CA**	**MI**	**TR**	**BV**
Kim et al. (428)	5,075	KOR	Adult patients	Disorder	6.98			•		
Bromet et al. (429)	3,231	USA	Disaster responders	Current	2.50					ø
Welsh et al. (234)	483	USA	Patients 11–24	Disorder	2.19					
Peltzer and Pengpid (430)	15,310	ZAF	Adolescents and adults	Daily	1.83			•		
Smith et al. (431)	3,119	USA	Military veterans	Disorder	1.59		•	•		
Xu et al. (432)	11,254	CHN	University students	Any	1.34	•				
Chou et al. (271)	36,309	USA	Adults 18+	Disorder	1.27			•		
Chou et al. (280)	36,309	USA	Adults 18+	Lifetime e-cig.	1.20		•	•		
Sawchuk et al. (279)	2,774	USA	American Indians 15–54	>100 cig.	1.15			•		
Whitworth et al. (433)	1,140	USA	Military veterans	Daily	1.12			•		
Ehlers et al. (423)	614	USA	Mexican Am. 18–30	Dependence	1.03					ø
Hruby et al. (281)	12,708	USA	Military service members	Past 30 days	0.97	•	•	•		
**Sedentary behavior (*****N*** = **4)**						**TO**	**CA**	**MI**	**TR**	**BV**
Kim et al. (196)	6,510	KOR	Adults 18–64	Addiction	2.09			•		
Whitworth et al. (433)	1,140	USA	Military veterans	Low PA	1.84			•		
Zhang et al. (197)	7,121	CHN	Adults 18–81	TV >3 h/day	1.28	•		•		
Hruby et al. (281)	12,708	USA	Military service members	PA < 75 min/week	1.22	•	•	•		
**Diet quality (*****N*** = **1)**						**TO**	**CA**	**MI**	**TR**	**BV**
Mutiso et al. (198)	9,742	KEN	High school students	Binge eating	1.04	•	•	•		
	**Median**	**Unweighted mean (SD)**		**Weighted mean**						
Cannabis	1.80	2.30 (1.47)		1.83						
Tobacco	1.31	1.93 (1.66)		1.56						
Sedentary behavior	1.56	1.61 (0.43)		1.47						
Diet quality	1.04	1.04		1.04						

OR, odds ratio; SD, sedentary behavior and diet quality; TO, tobacco; CA, cannabis; MI, social misery; TR, psychological trauma; BV, bivariate (unadjusted).

**Table 7b:** Longitudinal PTSD studies (*N* = 2).

**Longitudinal: behavior before disorder (*****N*** = **2)**					**Covariate adjustments**					
**Cannabis (*****N*** = **1)**	***N***	**Area**	**Sample**	**Outcome**	**OR**	**SD**	**TO**	**MI**	**TR**	**BV**
Lee et al. (434)	674	USA	Adolescence->adulthood	Weekly	4.68		•		•	
**Tobacco (*****N*** = **1)**						**SD**	**CA**	**MI**	**TR**	**BV**
Ibrahim et al. (435)	7,561	USA	Adults 18+	Dependence	1.59		•	•	•	
	**Median**	**Unweighted mean**		**Weighted mean**				
Cannabis	4.68	4.68		4.68				
Tobacco	1.59	1.59		1.59				

OR, odds ratio; SD, sedentary behavior and diet quality; TO, tobacco; CA, cannabis; MI, social misery; TR, psychological trauma; BV, bivariate (unadjusted).

### Analyses

#### Aim 1: understand individual associations between a given unhealthy behavior and a given mental disorder in relation to other such disorders

**Figure 2:**
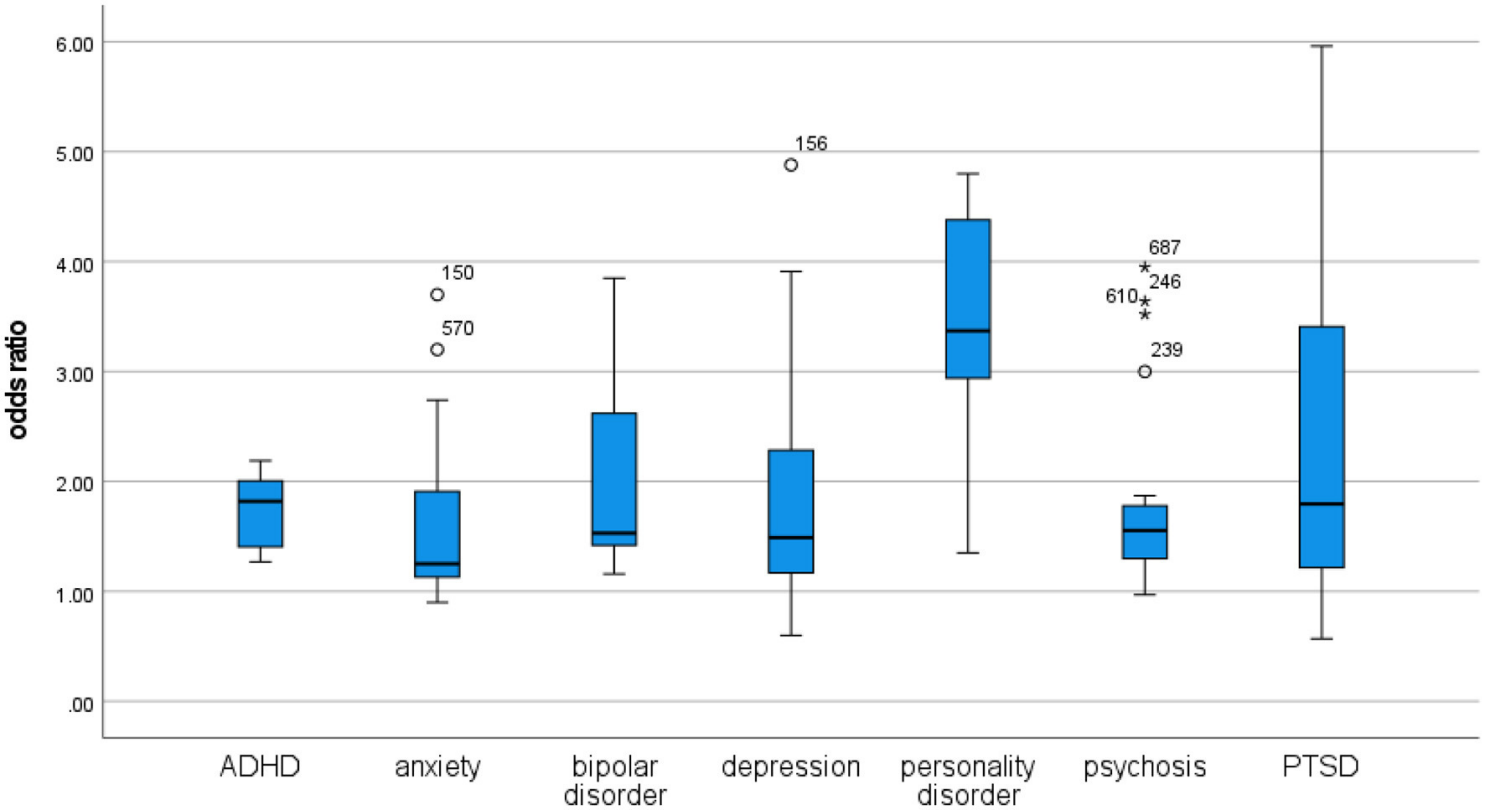
Odds ratios for cross-sectional studies of cannabis and psychiatric disorders.

The general finding of this review was that associations between a given unhealthy behavior and mental disorders tended to resemble each other in terms of their strength (Figure 2 and Supplementary Figures A2a–A2d), although with some exceptions. In studies of tobacco use, associations with psychosis tended to be stronger than associations with other mental disorders, with the differences reaching statistical significance for cross-sectional studies in *t*-test comparisons with depression (*t* = 2.34, df = 88, two-sided *p* = 0.022), anxiety (*t* = 2.83, df = 24.07, two-sided *p* = 0.009), and ADHD (*t* = 2.09, df = 28, two-sided *p* = 0.046). Associations with anxiety for their part tended to be weaker than associations with other mental disorders, reaching statistical significance for cross-sectional studies in *t*-test comparisons with personality disorder (*t* = 2.61, df = 33, two-sided *p* = 0.014) and marginal significance (*t* = 1.86, df = 97, two-sided *p* = 0.066) with depression. In studies of sedentary behavior and diet quality, associations with PTSD tended to be weaker than associations with other mental disorders, although low numbers of studies for many disorders entailed that the differences reached marginal statistical significance only in cross-sectional *t*-test comparisons with psychosis (*t* = 2.03, df = 9, two-sided *p* = 0.07).

Because of constraints on space, it is not possible to report more detailed analyses for all the behaviors here reviewed. However, since the association between cannabis use and psychosis was previously noted as especially controversial, it will be analyzed in further detail. Two analyses compared the strength of the cannabis-psychosis association with that of associations between cannabis use and other mental disorders. The first such analysis compared the means and spreads for each disorder between different studies, with the boxplots of Figure 2 summing up this analysis for cross-sectional studies of cannabis use. These studies indicate that cannabis use was not more strongly associated with psychosis than with the other mental disorders here under purview. *T*-tests indicated that only the figures for personality disorder were significantly different from those for psychosis (*t* = 4.18, df = 27, two-sided *p* < 0.001), and indeed the figures for personality disorder were significantly higher than those for all other diagnoses (depression *t* = 4.33, df = 50, two-sided *p* < 0.001; anxiety *t* = 5.44, df = 30, two-sided *p* < 0.001; bipolar disorder *t* = 2.73, df = 16, two-sided *p* = 0.015; ADHD *t* = 4.44, df = 9.66, two-sided *p* < 0.001; PTSD *t* = 2.13, df = 25, two-sided *p* = 0.043). In addition, the association with anxiety was marginally weaker than the association with PTSD (*t* = 1.78, df = 24.05, two-sided *p* = 0.06). No differences were detected for longitudinal studies or specifically for longitudinal (behavior before disorder) studies (Supplementary Figures A2a, A2b), but there were not many such studies to compare.

As can be seen in Tables 1a–7a, furthermore, the weighted means of odds ratios for cross-sectional associations between cannabis use and the seven mental disorders tended to converge even more closely than the unweighted means to the area of 1.5–2.0, with the only exception relating to personality disorders. Longitudinal means (Tables 1b–7b) were somewhat more varied, but the weighted means for longitudinal (behavior before disorder) studies converged to the area of 1.0–2.0 except for ADHD, for which there was no evidence, and for a single high-OR PTSD study. Weighted means for the small number of longitudinal (disorder before behavior) studies for their part converged to the area of 0.5–1.5.

The second analysis focused on within-study comparisons. Some of the studies included in this review reported comparable figures for associations between cannabis use and psychosis as well as other disorders, and these figures are presented in Table 8 with an OR cut-off of 0.25. Longitudinal results related to having a mental disorder at baseline and subsequent initiation of cannabis or tobacco use have been ignored as they are not relevant for the comparison of putative harms resulting from the behavior. Unfortunately, only a few studies reported comparable figures, and there were no figures at all for ADHD and personality disorder. According to the available data, however, the strength of the association between cannabis use and psychosis seems to be at the same level as the strength of the associations between cannabis use and depression, anxiety, bipolar disorder, and PTSD. It appears that no reviews have investigated the associations between cannabis and psychosis as well as cannabis and other disorders at the same time.

**Table 8:** Cannabis-psychosis compared to cannabis-other disorders.

	**Depression**	**Anxiety**	**Bipolar disorder**	**PTSD**
Psychosis stronger (OR + 0.25)	Campbell et al. (176)	Campbell et al. (176)	Campbell et al. (176)	Campbell et al. (176)
Psychosis and other disorders on same level	Chan et al. (183) Davies et al. (171) Hines et al. (182)	Chan et al. (183)		
Other disorders stronger (OR + 0.25)	Padwa et al. (151)	Hines et al. (182) Padwa et al. (151)	Padwa et al. (151)	Padwa et al. (151)

It is worth mentioning that the study by Padwa et al. (151) found that cannabis use was also associated with a broad range of general medical disorders including circulatory system disease, digestive system disease, and musculoskeletal disease. The strength of these associations was broadly at the same level as the strength of the associations with mental disorders.

#### Aim 2: understand individual associations between a given unhealthy behavior and a given mental disorder in relation to other such behaviors

**Figure 3:**
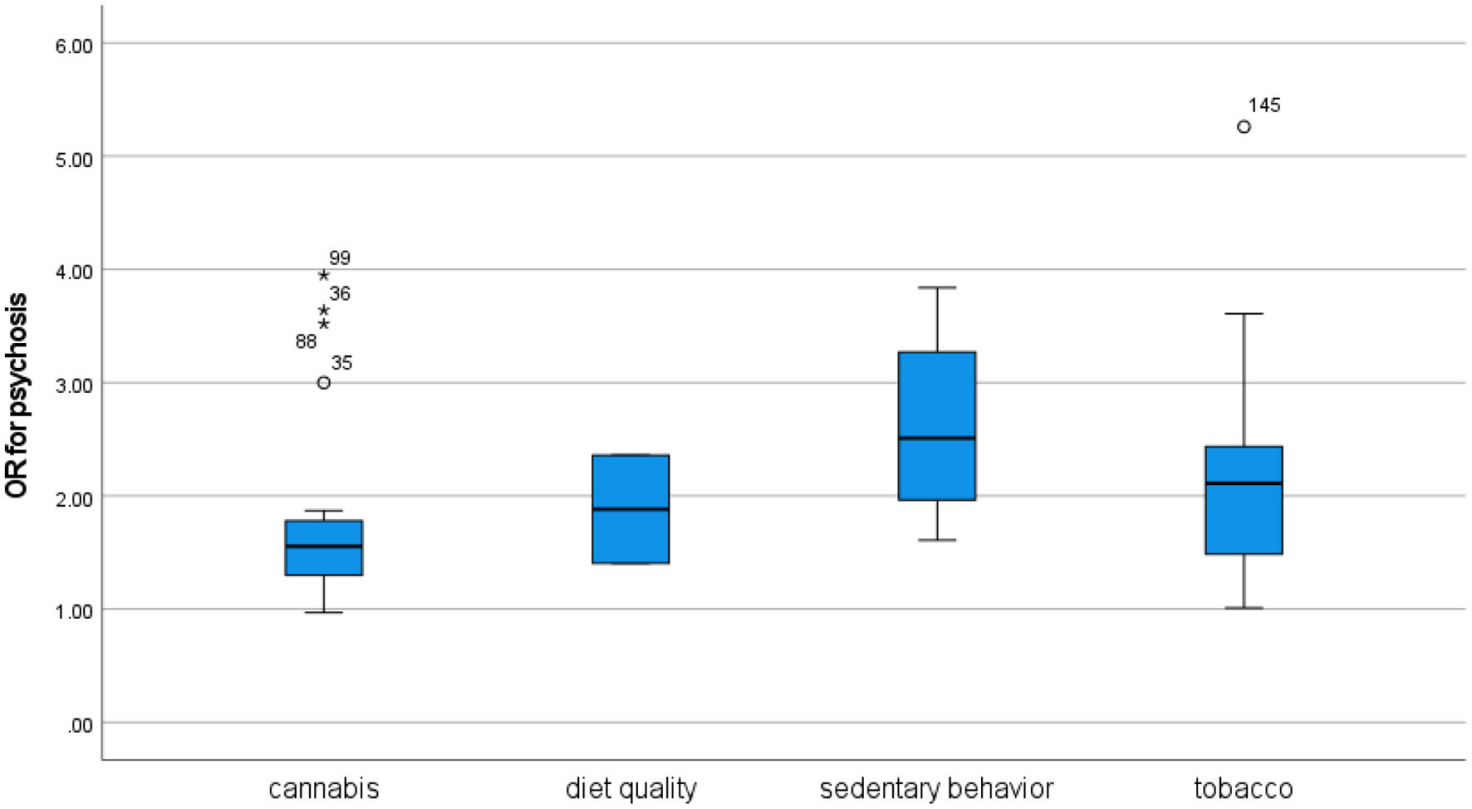
Odds ratios for psychosis in cross-sectional studies.

**Figure 4:**
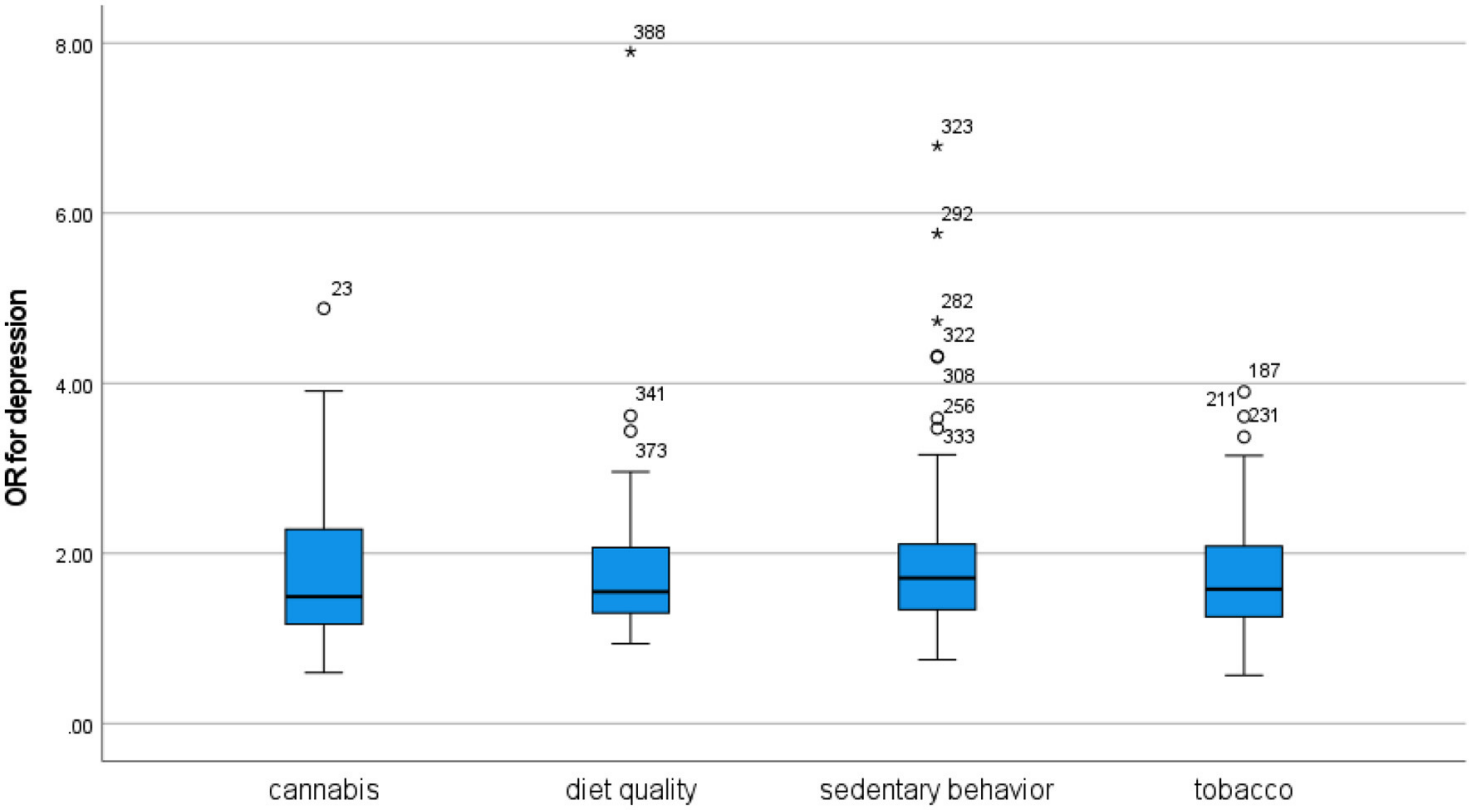
Odds ratios for depression in cross-sectional studies. In order to improve the quality of presentation, one outlier study (152) with a very high OR of 28.19 for diet quality and depression has been removed.

**Figure 5:**
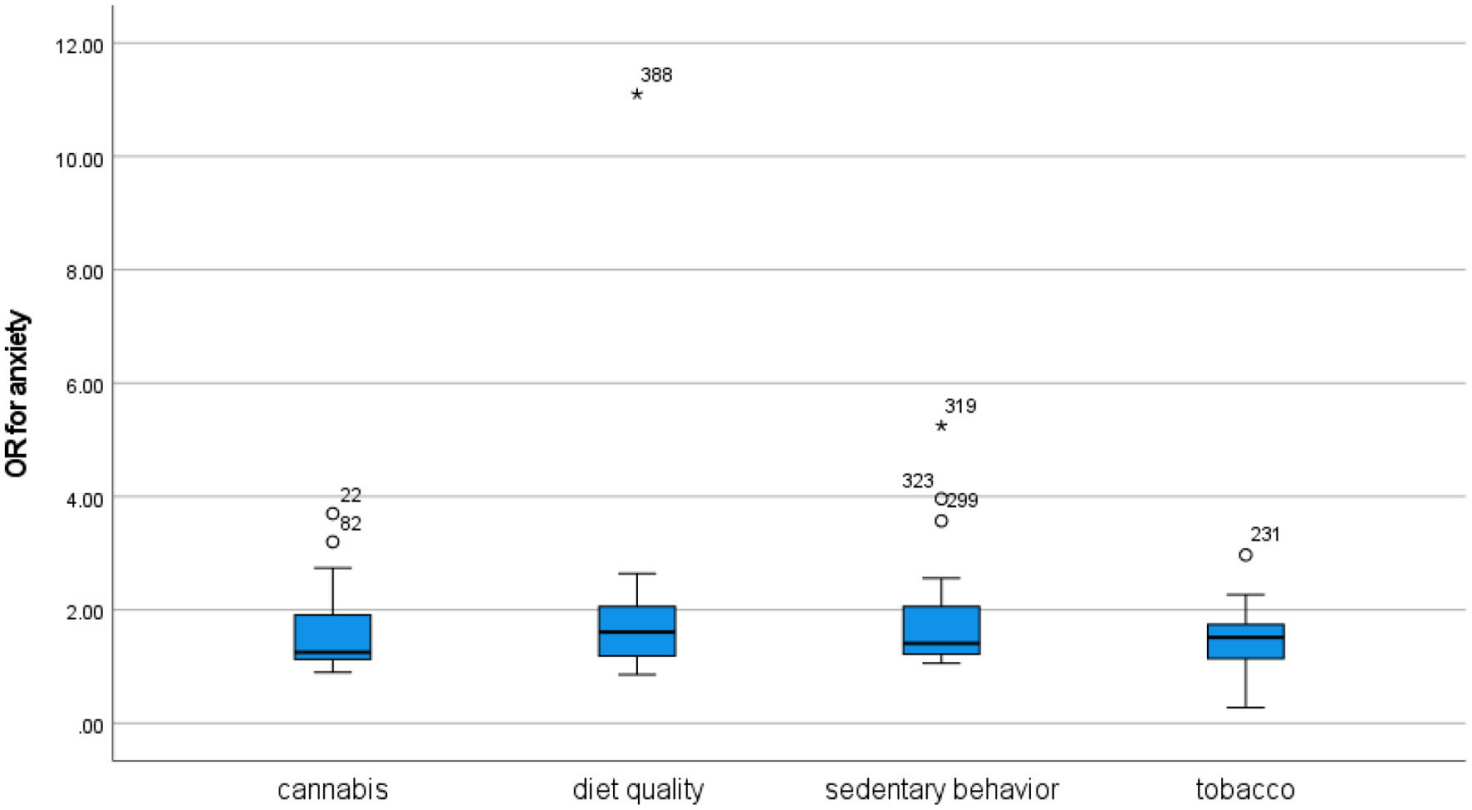
Odds ratios for anxiety in cross-sectional studies.

**Figure 6:**
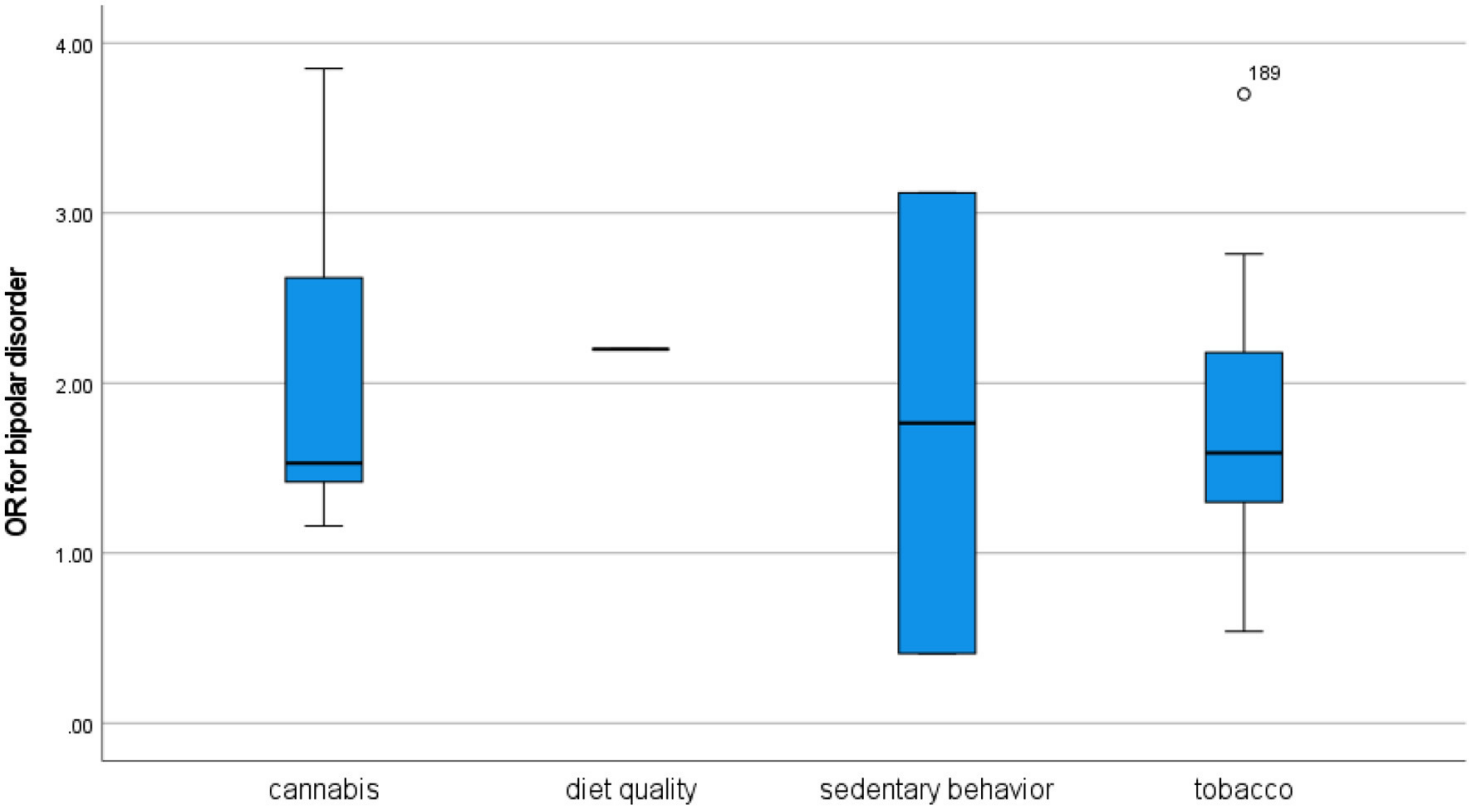
Odds ratios for bipolar disorder in cross-sectional studies.

**Figure 7:**
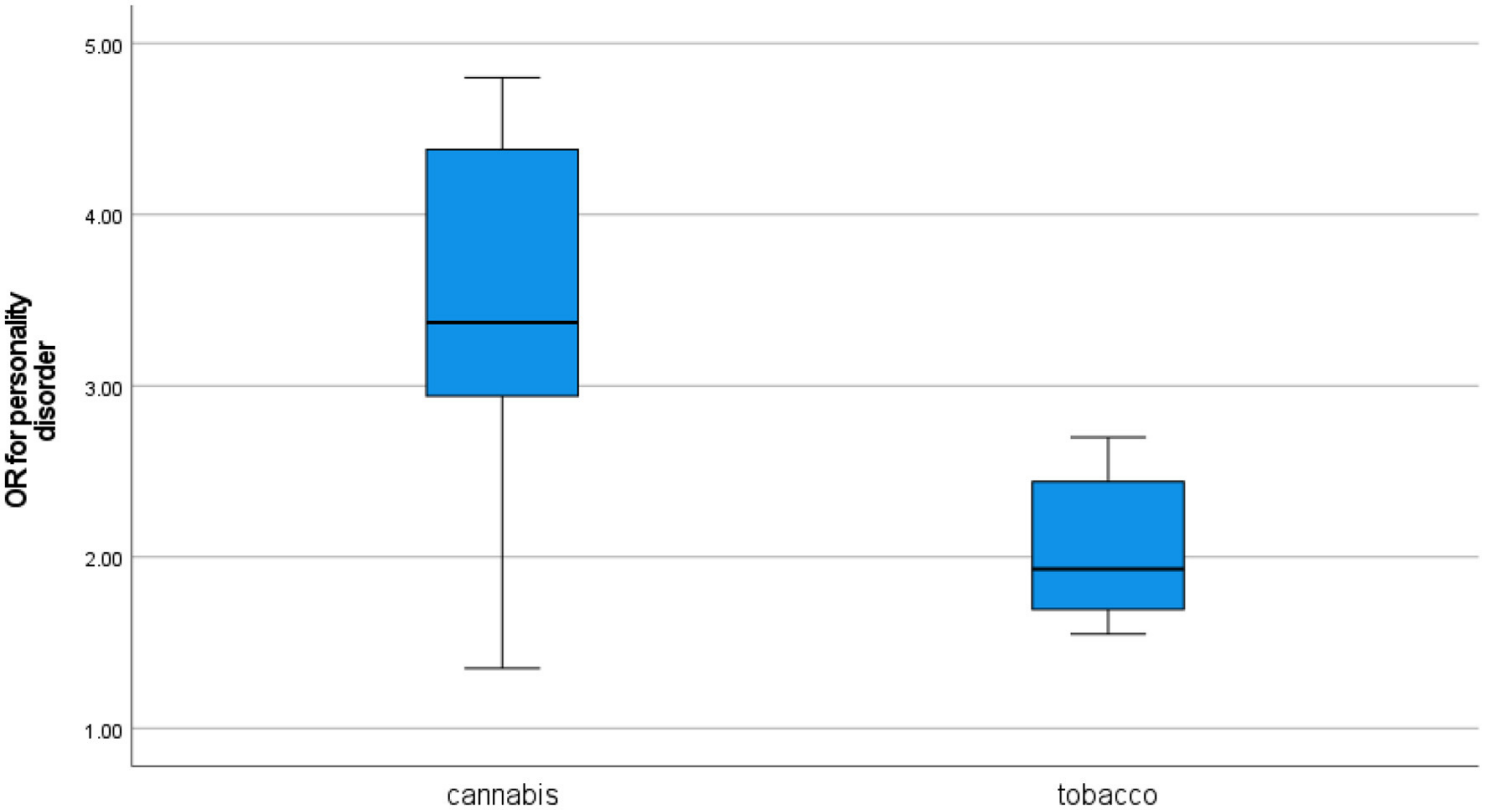
Odds ratios for personality disorder in cross-sectional studies.

**Figure 8:**
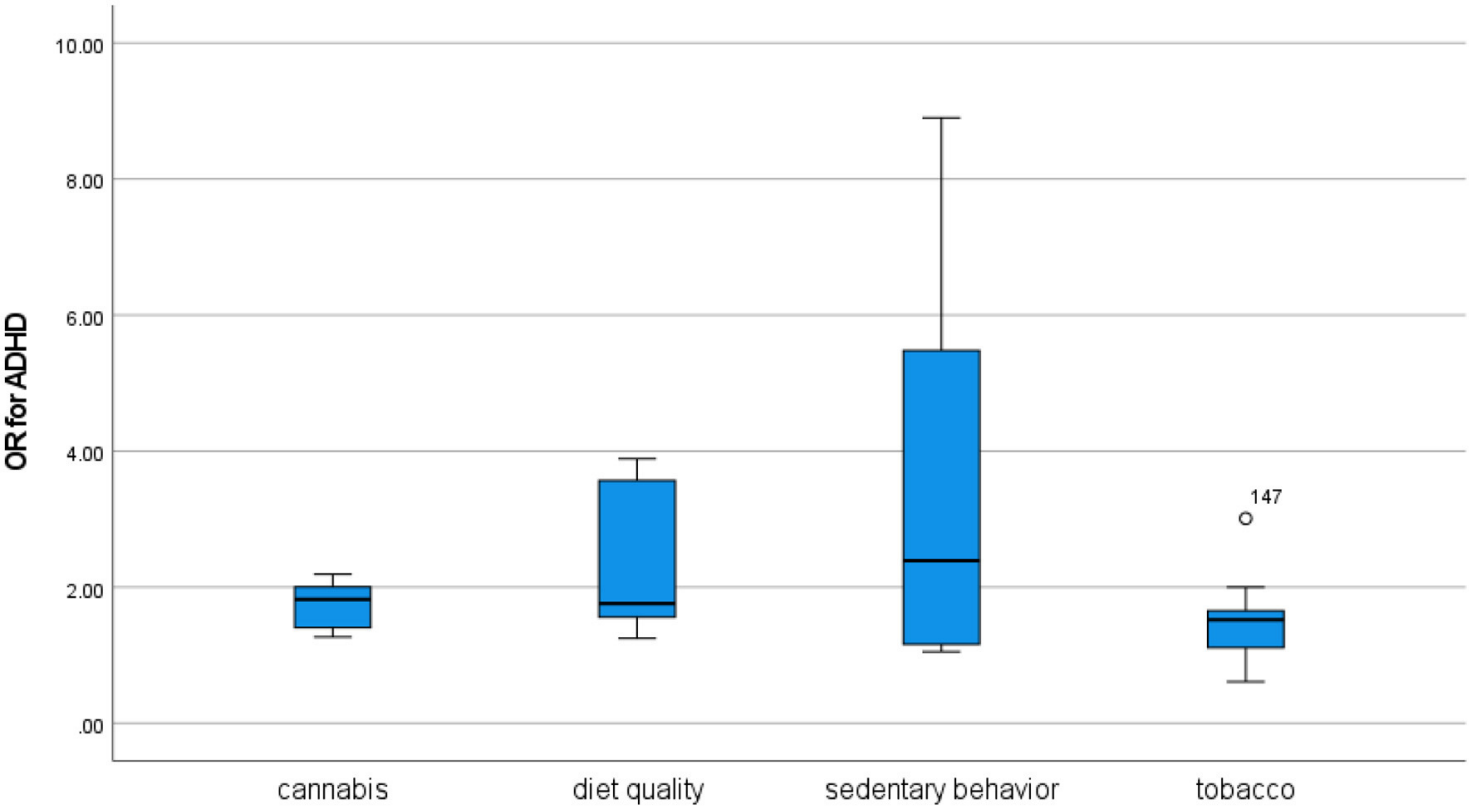
Odds ratios for ADHD in cross-sectional studies.

**Figure 9:**
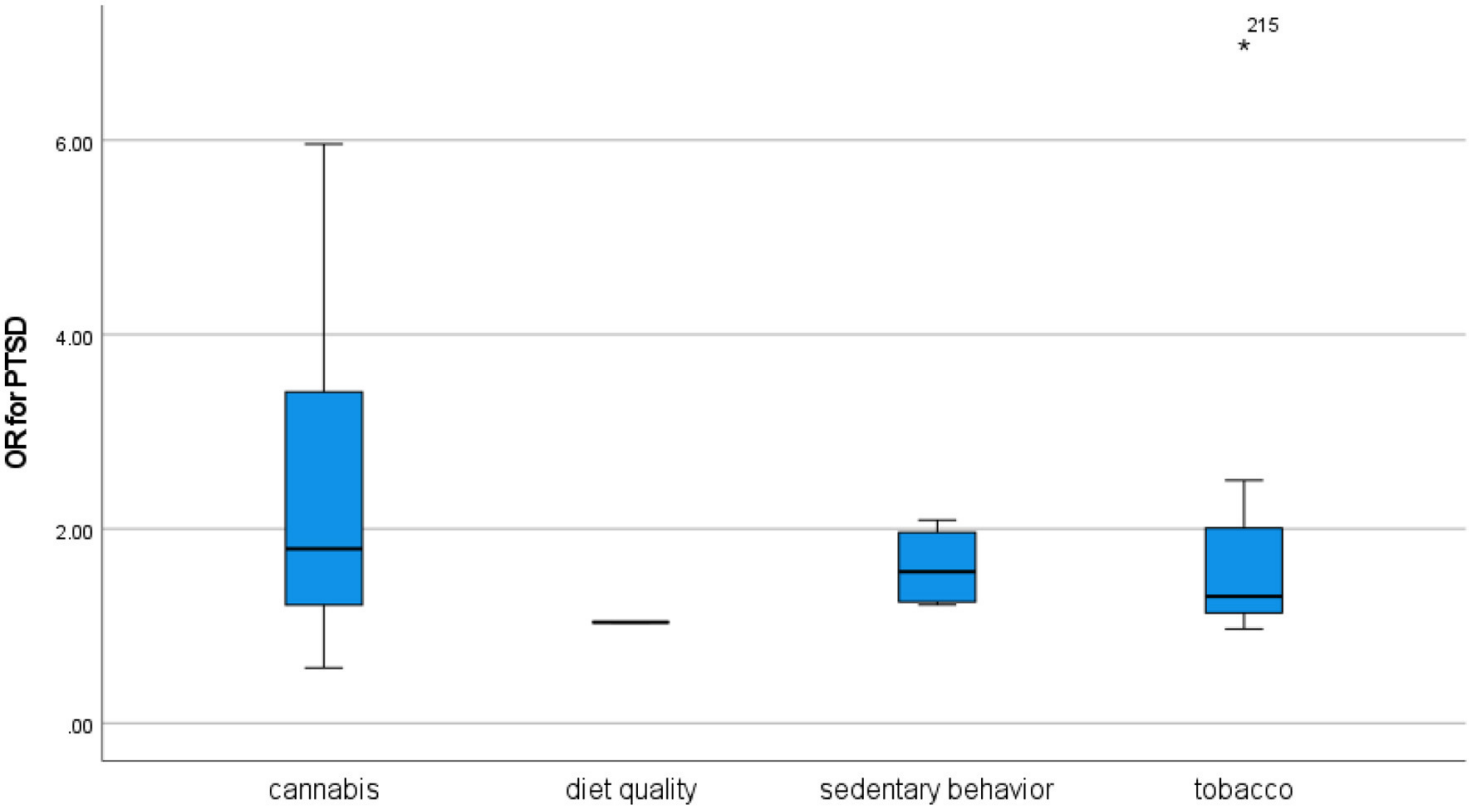
Odds ratios for PTSD in cross-sectional studies.

Two analyses compared the strength of associations between different unhealthy behaviors and a given mental disorder. The first such analysis compared the means and spreads for each disorder between different studies. The boxplots of Figures 3–9 sum up this analysis for each disorder in cross-sectional studies, while boxplots for longitudinal (behavior before disorder) studies are available in the Supplementary Figures A3–A6. Note that in order to improve the quality of presentation, one outlier study (152) with a very high OR of 28.19 has been removed from the figures for depression.

For the overall comparisons between cannabis and tobacco, *t*-tests indicated that only the figures for personality disorder were significantly different from each other (*t* = 3.13, df = 4, two-sided *p* = 0.007) in cross-sectional studies. There were no significant differences for longitudinal (behavior before disorder) findings. There were also no significant differences between cannabis and diet quality studies, although the number of studies available for each analysis was sometimes very small. For comparisons between cross-sectional studies of cannabis and sedentary behavior, *t*-tests indicated that the figures for ADHD (*t* = 1.83, df = 9.33, two-sided *p* = 0.099) were marginally significant. Tobacco use tended toward lower-strength associations with mental disorders than sedentary behavior and poor diet quality in cases where there were enough studies to make meaningful comparisons. These comparisons (with sedentary behavior and diet quality combined) reached significance for ADHD (*t* = 2.56, df = 19.94, two-sided *p* = 0.019). There were no significant differences between sedentary behavior and diet quality.

Weighted means gave emphasis to a single large-*N*, high-OR study of sedentary behavior and psychosis that made the cross-sectional association between sedentary behavior and psychosis appear to be substantially stronger especially than the association for cannabis. The weighing also gave emphasis to a single large-*N* study of sedentary behavior and bipolar disorder that made sedentary behavior seem protective against this disorder. Weighted means otherwise supported a difference between cannabis and tobacco with regard to personality disorder, but tended to mitigate the differences between cannabis use and sedentary behavior with regard to ADHD and PTSD. For longitudinal studies, weighing tended to reduce differences in odds ratio means between the different behaviors.

The second analysis focused on within-study comparisons. Some of the studies included in this review reported comparable figures for associations between different behaviors and a specific mental disorder, and these figures are presented in Table 9 with an OR cut-off of 0.25. In order to qualify as comparable, figures should reflect light, moderate or high use of both cannabis and tobacco, although exceptions were made for cases where differences in association strength were maintained despite a divergence in behavior intensity that presumably weakened the strongest association. In Magklara et al. (153), for instance, ever-use of cannabis incurred a higher OR for depression than daily use of tobacco, and this finding has been added to Table 9 despite the sub-moderate threshold for inclusion in the cannabis group. Longitudinal results related to having a mental disorder at baseline and subsequent initiation of cannabis or tobacco use have been ignored as they are not relevant for the comparison of putative harms resulting from the behavior.

**Table 9:** Associations between cannabis and psychiatric disorders compared to tobacco.

	**Psychosis**	**Depression**	**Anxiety**
Cannabis stronger (OR + 0.25)	Bhavsar et al. (172) Davies et al. (171) Degenhardt et al. (121) Jones et al. (200)	Cougle et al. [(213); CS] Davies et al. (171) *Esmaeelzadeh et al*. (18) Magklara et al. (153) Mannes et al. (217) Matta et al. (150) Porras-Segovia et al. (114) Risal et al. (208)	Risal et al. (208) Welsh et al. (234)
Cannabis and tobacco on same level	Carney et al. (179) Degenhardt et al. (202) McMahon et al. (120)	Chadi et al. (218) Estévez et al. (221) Wang and Peiper (228) Welsh et al. (234)	Cougle et al. [(213); CS] López-Gil et al. (363)
Tobacco stronger (OR + 0.25)	Ferraro et al. (177) Fusar-Poli et al. (8) Ryan et al. (203)	Prestage et al. (223) Seaman et al. (226) Tiburcio Sainz et al. (235)	*Esmaeelzadeh et al*. (18) Mannes et al. (217) Prestage et al. (223)

Meta-analyses indicated by italics.

CS, cross-sectional; LN, longitudinal.

We see in Table 9 that cannabis and tobacco appear to be associated with psychosis and anxiety at about the same level. For depression, the association for cannabis appears to be slightly stronger than that for tobacco. Other disorders were less commonly studied, but Supplementary Table A9 indicates that cannabis may be more strongly associated with personality disorder and perhaps ADHD than tobacco is, while the very limited evidence available indicates that their association with bipolar disorder and PTSD is about equally strong.

Table 10 compares diet quality and sedentary behavior to cannabis and (most commonly) tobacco use. Depression is fairly well-studied, and it appears that the association with diet quality and sedentary behavior is at the same level as the association with cannabis/tobacco use. Less evidence is available for psychosis and anxiety, but the evidence for psychosis seems to indicate that the association with diet quality and sedentary behavior is at least as strong as the association with cannabis/tobacco use, while the opposite may be true for anxiety. Very limited evidence for ADHD and PTSD indicates that the associations with diet quality and sedentary behavior are as strong or stronger than the associations with cannabis/tobacco use (Supplementary Table A10).

**Table 10:** Associations between cannabis/tobacco and psychiatric disorders compared to diet quality and sedentary behavior.

	**Psychosis**	**Depression**	**Anxiety**
Cannabis/tobacco stronger (OR + 0.25)		Albasara et al. (130) Kim et al. (264) López-Sánchez et al. (133) Ma et al. (246) Masana et al. (261) Rahe et al. (245) Wang et al. (244)	Hruby et al. (281) López-Sánchez et al. (133)
Cannabis/tobacco and diet quality/sedentary behavior on same level	Werneck et al. [(189); DQ]	Cabello et al. (352) Hruby et al. (281) Kim [(263); DQ] Melin et al. (255) Nam et al. (131) Peltzer and Pengpid (272) Tsutsumimoto et al. (351)	López-Gil et al. (363)
Diet quality/sedentary behavior stronger (OR + 0.25)	*Fusar-Poli et al*. (8) McMahon et al. (120) Werneck et al. [(189); SB]	Kim [(263); PA] Liu et al. (265) Luo et al. (258) Pengpid and Peltzer (276) Wang and Peiper (228) Zhang et al. (350) Zhu et al. (282)	

Meta-analyses indicated by italics.

DQ, diet quality; PA, physical activity; SB, sedentary behavior.

## Discussion

### Aim 1: understand individual associations between a given unhealthy behavior and a given mental disorder in relation to other such disorders

According to the findings of this comparative review, the association between a given behavior and a given disorder tended to resemble similar associations with other disorders. Some disorders distinguished themselves as particularly weakly or strongly associated with a given behavior, such as psychosis and anxiety for tobacco use and PTSD for sedentary behavior and diet quality, but even in these cases the differences were of moderate magnitude. Taking the cannabis-psychosis association as a basis for comparison, it appears that cannabis use is not more strongly associated with psychosis than with the other disorders. Comparisons across studies and within studies both supported this conclusion. However, personality disorder appeared to be more strongly associated with cannabis use than what is the case for psychosis and other disorders. Furthermore, in some of the sensitivity analyses that removed specific groups of studies from the dataset, cannabis use appeared to be more strongly associated with PTSD than with psychosis. Thus, in contrast to the consensus paper by D'Souza et al. (1), which used a single study by Starzer et al. (137) as the basis for inferring that “[c]annabis is more likely to be associated with psychosis outcomes than other psychiatric diagnoses” (p. 732), this review found that cannabis use is equally strongly associated with a range of different mental disorders, with only personality disorder (and perhaps PTSD) distinguishing themselves in terms of being especially strongly associated with cannabis use. D'Souza et al.'s (1) identification of the specificity of the cannabis-psychosis relationship as an indication that this relationship may reflect a causal effect from cannabis use, as per the classic criteria by Hill (112), is therefore unsupported by the present review.

This conclusion should not be especially susceptible to variation in behavior intensity, as moderate (weekly) cannabis use was emphasized in findings across different disorders. Nevertheless, sensitivity analyses did identify disparities related to sample groups, behavioral measurement, and control variables that may have impacted findings. Within-study analyses should be immune to such effects as the studies included in these analyses utilized the same participant samples and control variables, however.

### Aim 2: understand individual associations between a given unhealthy behavior and a given mental disorder in relation to other such behaviors

The general finding from this review was that the association between a given behavior and a given disorder tended to resemble similar associations for other behaviors. Thus, it appears that moderate use of cannabis is about as strongly associated with mental disorders as is moderate use of tobacco and high use of fast food, sugary beverages, and television. In terms of their respective behavior intensity, conceptualized as the behavior related to the median-area odds ratio in cross-sectional studies, cannabis use ranging from any exposure via weekly or current use to use disorder appears to be about as harmful as tobacco use ranging from any exposure via daily, regular or current use to use disorder. Both of these, in turn, appear to be about as harmful as behavior ranging from 2 to 4 h of daily screen time, 4 to 10 h of daily inactivity, or daily ingestion of sweets, sodas, and fast food up to binge eating or the disordered use of computer games, Facebook or the internet. The only statistically significant divergence at the 0.05 level related to personality disorder, which was more strongly associated with moderate cannabis use than with moderate tobacco use.

Within-study comparisons generally supported this conclusion, although with some variation for depression and ADHD. Thus, in contrast to the consensus paper by D'Souza et al. (1) that drew the inference that “[t]he risk for a psychosis outcome is highest for cannabis relative to other drugs” (p. 732), this review found that the association between cannabis use and psychosis is about equally strong as the association between psychosis and tobacco use as well as the associations with sedentary behavior and poor diet quality. It should be acknowledged, however, that the latter two seem not to have been intensively studied, and that variation in behavior intensity may have affected overall findings. This review emphasized moderate cannabis and tobacco use, operationalized as weekly cannabis use and daily tobacco use of around 10 cigarettes, as compared to high levels of sedentary behavior and poor diet quality. If the review had instead focused on high levels of cannabis use, the comparison would probably have favored tobacco use, sedentary behavior, and poor diet quality as less harmful behaviors.

There are, however, several reasons to focus on moderate (weekly) rather than high (daily or more) cannabis use in harms assessments. Heavy use of intoxicants typically reflects motivations related to coping, and such motivations are normally related to underlying problems that are themselves associated with risk for mental disorder (13). Furthermore, the frequent use of intoxicants may exacerbate such underlying problems via social mechanisms that are not directly related to the pharmacological effects of the intoxicant itself. A person who engages in frequent daytime intoxicant use will incur a range of negative consequences in terms of employment and social relations irrespective of whether the intoxicant is cannabis, alcohol, or some other substance, and these negative consequences will often exacerbate the underlying problems that motivate the frequent intoxicant use in the first place. Studies of such behaviors will therefore tend to conflate negative consequences incurred via social mechanisms and negative consequences incurred via pharmacological mechanisms. In order to avoid such conflation of effects and constrain the measurement of consequences toward those that are of a pharmacological nature, it would be advisable to focus on moderate intoxicant use.

The findings of this review could be understood in relation to Nutt's (154) comparison of the harms related to MDMA use and horse riding. Both studies related the risks incurred by substance use to risks incurred by ordinary and socially acceptable activities, and found that despite their negative reputation, use of substances such as MDMA, cannabis, and tobacco do not appear to be more harmful than these ordinary activities.

### Aim 3: understand the association between unhealthy behaviors and mental disorders in relation to statistical control for covariates

This review constructed dichotomous indicators for covariate adjustment in order to better understand the overall impact of such adjustment on studies of associations between unhealthy behaviors and mental disorders. The most influential of these adjustment indicators were those for cannabis and tobacco use, which reached significance at the 0.05 level in overall correlation with odds ratios across disorders in non-cannabis and non-tobacco studies, respectively. This finding allows for the conclusion that failing to control for cannabis and tobacco use, both of which tend toward collinearity both with each other and with other unhealthy behaviors as well as with a number of mental disorders, entails an exaggeration of the strength of the association under scrutiny. In other words, failing to control for cannabis and tobacco use in studies of unhealthy behaviors and mental disorders may amount to analytical malpractice. At a minimum, such studies should report comparable figures for cannabis and/or tobacco so that readers may calibrate the results presented for the unhealthy behavior under scrutiny in comparison to figures for cannabis and tobacco use.

The indicator for adjustment for diet quality and/or sedentary behavior was not correlated with odds ratios in cannabis and tobacco studies, but the inclusion of such adjustment was also so uncommon that the overall statistics of its impact may not be trustworthy. This applies especially to cannabis, as only 1% of cannabis studies controlled for diet quality and/or sedentary behavior and only 8% of studies of the latter behaviors controlled for cannabis use. It thus appears that researchers tend to understand cannabis use and poor diet quality/sedentary behavior as conceptually distinct, with cannabis use constituting a form of very harmful drug use whereas frequent consumption of fast food or television merely constitutes an unhealthy lifestyle choice. The findings of this review indicate that any such distinction, at least with regard to moderate cannabis use, is probably illusory, as weekly cannabis use appears to be about equally harmful as weekly to daily consumption of cheeseburgers.

Tobacco use appears to be positioned somewhat more closely to poor diet quality/sedentary behavior on a conceptual level. A majority (55%) of studies of the latter behaviors controlled for tobacco use, and the 14% of tobacco studies which controlled for diet quality and/or sedentary behavior at least outnumber the corresponding number of cannabis studies (1%) by a wide margin. Thus, there is some evidence of a tendency among researchers to understand tobacco use as a form of unhealthy behavior that is conceptually related to other unhealthy behaviors that do not involve substance use.

The indicator for bivariate analysis was also fairly influential on overall odds ratios, although perhaps not to the extent that one would expect. Only a small number of studies reported bivariate figures, and in comparative analyses with many included studies we would expect the influence from bivariate figures to cancel each other out on average. Sensitivity analyses that removed the studies with bivariate figures from the dataset introduced differences, albeit of marginal significance, relating to the association between cannabis use and PTSD. It would therefore appear that studies investigating the cannabis-PTSD relationship were especially impacted by figures lacking statistical control.

Indicators for trauma and misery adjustment had some influence on overall odds ratios and localized impact of a more substantial nature on findings related to specific disorders and behaviors. It is therefore true to say that the methods used to analyze the impact from trauma and misery on the studies included in this review did not identify a major impact on association strengths. Nevertheless, since this review focused on behaviors of moderate intensity for tobacco and cannabis use, the finding of a tentative or light influence could be said to reflect expectations. Moderate use was here conceptualized as use driven by a broad range of motivations, in contradistinction to the heavy use understood as being more often driven by coping motivations related to underlying trauma and misery. As such, one would expect that indicators for trauma and misery adjustment would have a more substantial impact on analyses of heavy substance use than what was the case in the present review.

It is important to note that if the association between substance use and mental disorder reflects, to some meaningful extent, an association between substance use and underlying factors of trauma and misery, then overall changes in the user population toward or away from more widespread use should be accompanied by corresponding changes to the strength of the association between the substance use and mental disorder. When the use of a given substance becomes more prevalent, at least in terms of large-scale changes, this normally reflects an acceptance of this substance among people who are driven primarily not by coping motives but by recreational, social, enhancement or expansion motives. Similarly, when the use of a previously prevalent substance is reduced, this normally reflects reduced acceptance of the given substance among people driven primarily by non-coping motives. In the western world, recent decades have seen reduced acceptance of tobacco use and increased acceptance of cannabis use, and we would therefore expect an increasing association between mental disorder and tobacco use along with a decreasing association for cannabis use.

These expectations are supported by research findings. Talati et al. (155) found that while overall tobacco use declined in the U.S. from the 1940s to 1980s, the proportion of smokers with nicotine dependence increased from 31% to 70% and the number of dependent smokers with depression, ADHD, bipolar disorder, and personality disorder increased significantly more than among non-smokers. In Canada, Yang and D'Arcy (156) similarly found that depression among older adults smokers increased substantially more than among non-smokers over the years 1994–2014. For cannabis, several studies have found increases in overall use without corresponding increases in cannabis use disorder (157–165), although there is also some conflicting evidence (166–168). Changes in associations with mental disorders have not been intensively explored, but one study by Livne et al. (169) found that “the association between cannabis use (but not CUDs) and dysthymia has weakened over time” (p. 327). If ongoing policy liberalization continues, the proportion of users with use disorder might be expected to further weaken, and the same could be said for the association between overall cannabis use and mental disorders.

### Limitations

Beyond the classification of specialty samples, behavioral measurement disparities, and statistical control for social misery and psychological trauma, this review has not assessed the methodological validity of the studies included. In particular, their utilization of healthy controls for patient samples and their construction of samples putatively representing the general population have not been assessed. Relatedly, the review has not accounted for variation in the measurement of mental disorders, which is a possible confounder to the comparative analyses. It is also possible that the focus on studies presenting their findings in terms of odds ratios may constitute a source of bias, although there is no particular reason to believe that findings presented as odds ratios should be stronger or weaker than findings presented as hazard ratios or in some other way.

The dichotomous indicator variables for trauma and misery adjustment constructed in this review might be criticized for being overly simplistic. It is possible that a more complex set of adjustment indicators would have been more sensitive for nuanced variations in covariate regimes in the included studies. A high percentage of these studies controlled for education, but although education is correlated with poverty and unemployment, adjustment for this single variable by no means exhausts the impact from social misery on unhealthy behaviors and mental disorders. Adjustment for psychological trauma was far less common, making a single dichotomous indicator more appropriate.

This review was limited to records from the Pubmed database published in 2015 or later. Such a selection of recent studies registered in Pubmed may have served to introduce bias in overall findings, although the author is not aware of any reason to believe that the Pubmed database is systematically different from other databases. As such, the present selection of studies could arguably be regarded as representative of the full population of such studies. Nevertheless, the present findings should be regarded as tentative and explorative until confirmed by more comprehensive investigations.

Furthermore, this review has analyzed and compared included studies at the level of their published findings rather than in terms of their underlying data. While this approach arguably yielded noteworthy results, it would undoubtedly be interesting to perform a comparative meta-analysis of the datasets for the behaviors and disorders here under purview. Such a meta-analysis would probably have to limit its scope as compared to the present review, but would allow for a more comprehensive understanding of the tentative findings obtained here. It should also be noted that the author of the present review performed all the assessments and analyses singlehandedly, increasing the risk of bias. Inter-rater reliability therefore could not be established. Moreover, the comparisons reported in this review were not Bonferroni corrected, and because 252 separate analyses were performed it is unlikely that any association would have maintained statistical significance after such correction.

Publication bias should be assumed to have affected the results presented in this review. Indeed, it was not infrequently the case that studies which investigated several relationships between unhealthy behaviors and disorders only reported odds ratios for those relationships that were statistically significant. Unless there is reason to believe that such bias should have affected some relationships substantially more than others, however, the comparative perspective of this review probably entails that publication bias on each side of the comparison would tend to cancel each other out.

It must be acknowledged that this review compared a broad range of studies across different populations and participant samples, although the same is true for more narrow reviews examining only one of these behavior-disorder relationships (17). Variation related to specialty samples, behavioral measurement disparities, and control variables did not appear to have a major impact on overall findings, which were largely robust across relevant sensitivity analyses. The findings should nevertheless be regarded as explorative and interpreted with caution. While further research is necessary in order to confirm the results obtained in this review, the apparent convergence of the strength of associations between four different types of unhealthy behaviors and seven different mental disorders is a noteworthy if tentative basis for further investigations.

A useful extension to the comparisons in this review would be to include studies of alcohol. As an intoxicant, alcohol may constitute a closer parallel to cannabis than the other unhealthy behaviors under scrutiny in this review, and it seems possible to compare the two in terms of both moderate use and heavy chronic use. It would also be interesting to extend such comparisons to other illicit drugs, although the associations between such drug use and mental disorder may not yet have been studied to such an extent that it is meaningful to include them in a comparative review.

## Conclusion

According to this review, the association between moderate cannabis use and mental disorder is about equally strong for psychosis, depression, anxiety, bipolar disorder, ADHD, and PTSD, while the less intensively studied association with personality disorder appears to be somewhat stronger than the others. Furthermore, these associations with cannabis use appear to be paralleled by associations with tobacco use, poor diet quality, and sedentary behavior at about the same strength, although once more with personality disorder as an exception. As such, the often-emphasized association between cannabis use and psychosis appears to lack specificity both across disorders and across behaviors.

When a broad range of different unhealthy behaviors appear to be about equally strongly associated with a broad range of mental disorders, we should probably understand them to reflect a general and shared etiology. The best candidate for a general explanation is arguably that this broad range of associations reflect underlying psychological trauma and social misery, which frequently appear together. Thus, the associations identified in this review may not represent a causal effect from the unhealthy behavior itself so much as a spurious relationship where both the behavior and the disorder are caused by underlying factors such as psychological trauma and social misery. Nevertheless, it should be noted that the analyses employed in the present review did not identify a major impact from variation in statistical control for such factors. Because of its exceptional strength, it is possible that the association between cannabis use and personality disorder constitutes an exception to this general explanation, although more research is required.

## Data availability statement

The original contributions presented in the study are included in the article/Supplementary material, further inquiries can be directed to the corresponding author.

## Author contributions

PJ: Conceptualization, Data curation, Formal analysis, Investigation, Methodology, Software, Validation, Visualization, Writing—original draft, Writing—review & editing.
