# Supplementary material for: Unhealthy behaviors associated with mental health disorders: a systematic comparative review of diet quality, sedentary behavior, and cannabis and tobacco use
Source: Front Public Health. 2024 Jan 5;11:1268339. doi: 10.3389/fpubh.2023.1268339 (PMC10797041; doi:10.3389/fpubh.2023.1268339)
Supplement: Supplementary file 2 [file Table_1.DOCX]

Appendices: Unhealthy behaviors associated with mental health disorders

# Appendix A – Coding issues for individual studies

## Alageel et al. (2021)

The study presented three different indicators for depression, “clinically significant depression” (elsewhere presumably referred to as moderate depression) (N = 40, OR 1.261), “moderately severe depression” (N = 53, OR 1.43), and “severe depression” (N = 42, OR 3.779). Weighted means were used in the review.

## Alenko et al. (2019)

The study also presented figures for tobacco use, but the figures appear to be erroneous and no reference group was provided. These figures have therefore not been included in the review.

## Apriliyasari et al. (2023)

For physical activity, the study reported an OR of 0.757 for meeting WHO criteria compared to not meeting criteria activity, which was inverted to an OR of 1.321 for not meeting WHO criteria.

## Bakhshaie et al. (2015)

Some of the information presented in the abstract appears to be erroneous. The review used the figure on persistent daily smoking and onset of depression.

## Bechtold et al. (2016)

The study stratified findings according to whether the adolescent boys had engaged in weekly cannabis use for 1-2 years (N = 3 with psychosis vs. N = 183 without) or 3+ years (N = 4 with psychosis vs. N = 55 without). Weighted means were used in the review.

## Bowling et al. (2017)

The study stratified findings according to whether children diagnosed with ADHD were medicated for the disorder (N = 380) or not (N = 270). Weighted means were used in the review.

## Butler et al. (2019)

The study’s final model controlled for a construct named flourishing, which included measurements of “life purpose and satisfaction, engagement and interest with daily activities, self-esteem and competence, and optimism to provide a score that represents overall psychological wellbeing” (p. 208). In order to obtain figures comparable to the other studies in the review, an exception was made for the general policy of using the most stringently controlled model available, and figures from the earlier model 1 was instead incorporated into the review.

## Carney et al. (2017)

For cannabis use, the study obtained ORs of 1.4 for current use, 1.2 for daily use, and 1.1 for problematic use. The review used the figure for current use, which seems to most closely approximate the moderate use aimed for here.

## Carrà et al. (2019)

The study stratified findings according to age group (N = 167338 adolescents with OR 1.61 and N = 360108 adults with OR 2.59). Weighted means were used in the review.

## Carriedo et al. (2020)

For physical activity, the study reported an OR of 0.774 for meeting vigorous physical activity guidelines, which was inverted to an OR of 1.29 for not meeting those guidelines.

## Chen et al. (2019)

The study stratified findings according to age group (N = 4445 adolescents with OR 2.2 and N = 13392 adults with OR 1.6). Weighted means were used in the review.

## Chido-Amajuoyi et al. (2021)

The study stratified findings according to the number of tobacco products used (single product 20.3%, OR 1.66; dual/poly product 9.7%, OR 3.09). To get an overall figure for use of any tobacco product(s), weighted means were used in the review.

## Chou et al. (2016)

The study differentiated between bipolar I and bipolar II and schizotypal, borderline, and antisocial personality disorder without indicating their relative prevalence. Unweighted means were used for bipolar disorder and personality disorder in the review.

## Chowdhury (2023)

The study provided ORs for the association between ADHD and 4-6 hours/day of screen time (N = 49; OR 7.5) and 6+ hours/day of screen time (N = 68; OR 7.11). Weighted means were used in the review.

## Claesdotter-Knutsson et al. (2021)

The study stratified findings according to gender and grade, with an OR of 1.15 for 9^th^ grade boys (N = 4056), an OR of 1.2 for 9^th^ grade girls (N = 4121), an OR of 1.51 for 2^nd^ grade boys (N = 3519), and an OR of 0.71 for 2^nd^ grade girls (N = 3682). Weighted means were used in the review.

## Clyde et al. (2015)

The study stratified daily smoking into “light smoking” (<10 cigarettes per day, prevalence 7.9%) and “moderate/heavy smoking” (11+ cigarettes per day, prevalence 12.6%). Weighted means were used in the review.

## Del-Ponte et al. (2019a)

The study stratified findings according to gender (N = 1489 boys, N = 1435 girls). Weighted means were used in the review.

## Elkins et al. (2018)

The study stratified findings according to gender (52% girls) and inattentive versus hyperactive-impulsive ADHD symptoms. Weighted means were used for gender and unweighted means for symptoms in the review.

## Fink et al. (2022)

The study stratified findings related to moderate CUD between borderline (N = 29, OR 1.64) and antisocial (N = 20, OR 0.94) personality disorder. Weighted means were used in the review.

**Formagini et al. (2017)**

The study stratified daily smoking into “light smoking” (<10 cigarettes per day, prevalence 47.4%) and “moderate/heavy smoking” (11+ cigarettes per day, prevalence 39.8%). Weighted means were used in the review.

## Galéra et al. (2017)

The study stratified daily smoking into <10 cigarettes per day (N = 2310) and 11+ cigarettes per day (N = 354). Weighted means were used in the review.

**Gomes et al. (2018)**

The study stratified findings according to gender (N = 508 men, N = 870 women). Weighted means were used in the review.

## Gorfinkel et al. (2022)

The study stratified findings according to grade (8^th^ grade N = 13041, OR 2.33; 10^th^ grade N = 13119, OR 1.76; 12^th^ grade N = 5636, OR 1.40). Weighted means were used in the review.

## Halladay et al. (2019)

The study stratified findings according to gender (49.9% men, 50.1% women). Weighted means were used in the review.

## Hasin & Walsh (2021)

This review provided an overview of NESARC results, from which those from NESARC-III have been included in the review. Findings for bipolar disorder were stratified according to subtype, and unweighted means were used in the review.

## Hasin et al. (2016)

The study stratified findings for bipolar disorder according to subtypes without indicating their relative prevalence. Unweighted means were used for bipolar disorder in the review.

## Holzer et al. (2022)

The study stratified findings according to age group (age 50-64, N = 17754, OR nicotine 1.87, OR cannabis 2.68; age 65+, N = 14011, OR nicotine 2.87, OR cannabis 3.27). Weighted means were used in the review.

## Hruby et al. (2021)

This study appears to use cross-sectional data to study bidirectional relationships between unhealthy behaviors and psychiatric disorders. The analyses reach similar but not identical results. The review used data from Table 3 in the original article and marked it as cross-sectional.

## Jiang et al. (2020)

For sedentary behavior, the study reported ORs of 0.28 for anxiety and 0.34 for depression related to the best category compared to the worst, which was inverted to ORs of 3.57 and 2.94 for the worst category compared to the best.

## Jones et al. (2018)

The study stratified findings according to early- and late-onset tobacco and cannabis use, with relevant prevalence being early-onset cigarette-only use (4.3%), early onset cannabis use (3.2%), late-onset cigarette-only use (14.8%), and late-onset cannabis use (11.9%). Weighted means were used in the review.

Whereas cigarette use was analyzed as cigarette-only, cannabis use was not separated from or controlled for cigarette use. As such, the figures for tobacco were controlled for cannabis use while the figures for cannabis were not controlled for tobacco use. The figures are therefore not directly comparable and have not been included in the within-study analysis.

## Kang et al. (2021)

For nutritional inadequacy, the study reported an OR of 0.698 for the top quartile as compared to the worst quartile, which was inverted to an OR of 1.433 for the worst quartile.

## Kerridge et al. (2018)

The study stratified findings according to gender weighted to represent the U.S. population, for which unweighted means were used in the review. The study also stratified findings related to bipolar disorder into bipolar I (prevalence 8.8% and 9.0% among male/female respondents with CUD) and bipolar II (prevalence 0.8% and 1.5% among male/female respondents with CUD). Weighted means were used for the figure for bipolar disorder in the review.

## Kim et al. (2022b)

The study stratified findings according to gender (N = 184514 men, N = 181891 women). Weighted means were used in the review.

## Lazarevich et al. (2018)

The study recruited 1131 students, but only reported OR figures for 615 females for which there were significant associations between depression scores and diet and physical activity.

## Leventhal et al. (2020)

The study stratified findings according to how many cannabis products were used (1 product: N = 208; 2 products N = 153; 3 products N = 99). Weighted means were used in the review. The study also stratified findings for ADHD according to inattentive and compulsivity symptoms, for which the unweighted mean was used.

## Li et al. (2022)

The study stratified findings according to study location (America N = 869, depression OR 1.14, anxiety OR 1.16; China N = 635 depression OR 0.94, anxiety OR 1.06). Weighted means were used in the review.

## Liu et al. (2016a)

The study stratified findings according to gender (N = 25920 men, N = 34282 women). Weighted means were used in the review.

## Livne et al. (2022)

The study presented separate findings from the NESARC (N = 43093, OR 3.70) and NESARC-III (N = 36309, OR 4.25). Weighted means were used in the review.

## López-Gil et al. (2022)

The study stratified findings for sedentary behavior according to gender (N = 14516 boys, N = 16872 girls). Weighted means were used in the review. The study did not report ORs for fast-food intake or tobacco use for boys or for cannabis use for girls.

## López-Sánchez et al. (2022)

The study stratified findings according to gender and form of impairment (vision or hearing). Weighted means of all four categories were used in the review.

## Mallet et al. (2018)

The study stratifies findings according to individual symptoms and in combinations of symptoms. The review used the most stringent model for current smokers vs. lifetime abstainers as associated with at least 5 symptoms.

## Matta et al. (2020)

The study stratified findings by gender and, for tobacco use, by low (1-9 cigarettes/day) and medium (10-19 cigarettes/day) use intensity. For cannabis, 325 men (OR 1.92) and 172 women (OR 1.37) had weekly or higher use. For tobacco, 1146 men (OR 1.12) and 1605 women (OR 1.23) smoked 1-9 cigarettes/day, while 989 men (OR 1.95) and 1099 women (OR 1.78) smoked 10-19 cigarettes/day. Weighted means were used in the review.

## McDowell et al. (2019)

For the prospective analysis of physical activity, the study reported an OR of 0.94 for meeting PA guidelines compared to not meeting guidelines, which was inverted to an OR of 1.06 for not meeting PA guidelines. For the cross-sectional analysis, the study similarly reported an OR of 0.91, inverted to 1.10.

## McMahon et al. (2021)

The study only reported bivariate figures for cannabis and tobacco use. The corresponding bivariate OR for maladaptive/pathological internet use was 5.92, which was the basis for the within-study comparison. The review otherwise used the OR of 2.70 for maladaptive/pathological internet use obtained from the multivariate regression model in McMahon et al.’s Table 4.

## Pengpid & Peltzer (2022)

The study stratified findings according to gender (N = 30577 men, N = 41685 women). Weighted means were used in the review.

## Porras-Segovia et al. (2018)

The study only reported bivariate figures for tobacco use, and the bivariate figures were used for the within-study comparison. The review otherwise used the OR obtained from the multivariate regression model.

**Prestage et al. (2018)**

The study only reported bivariate figures for cannabis use, and the bivariate figures were used for the within-study comparison. The review otherwise used the OR obtained from the multivariate regression model.

## Raffetti et al. (2019)

The description of the sample size in the main text of this this study (step 3, p. 698) does not seem to entirely match the information provided in Table 4. The review used the figures provided in Table 4.

## Sawchuk et al. (2016)

The study stratified findings according to tribe (Northern plains tribe, N = 1506, Southwest tribe, N = 1268). Weighted means were used in the review.

## Song et al. (2019)

The study presented results for active and secondhand smoking both before and during pregnancy. Exposure before pregnancy was used in the review.

## Sze et al. (2021)

The study stratified findings according to gender (men N = 201, OR depression 10.1, OR anxiety 1.13; women N = 223, OR depression 44.5, OR anxiety 0.86). Weighted means were used in the review.

## van Binnendijk et al. (2020)

The study stratified findings according to ethnicity, with 4580 native Dutch participants (OR 2.44), 3259 South Asian Surinamese (OR 1.83), 4292 African Surinamese (OR 1.75), 2262 Ghanaian (no significant observation observed and no OR reported), 3891 Turkish (OR 2.21), and 4187 Moroccan (OR 2.58). Weighted means were used in the review.

## Wang et al. (2021)

For diet quality, the study reported an OR of 0.848 for average Healthy Eating Index score, which was inverted to an OR of 1.18 for inadequate HEI.

## Weissenberger et al. (2018)

For tobacco use, the study reported an OR of 1.47 for no use compared to current use, which was inverted to an OR of 0.68 for current tobacco use. The study also reported an OR of 0.59 for a four-category cannabis variable but did not explain what this figure referred to, and it was not included in the review. Furthermore, the study reported an OR 0f 0.95 for a continuous TV variable measured in hours. This figure is not easily compared to other figures in the review and was not included.

## Wen et al. (2022)

For screen time, the study reported an OR of 0.391 for use less than 2 hours/day compared to use more than 2 hours/day, which was inverted to an OR of 2.558 for screen time >2 hours/day.

## Werneck et al. (2018)

The study stratified findings according to gender (49.3% girls). Weighted means were used in the review.

## Werneck et al. (2020a)

The study stratified findings according to gender (48454 boys, 52194 girls). Weighted means were used in the review.

## Werneck et al. (2021)

The study stratified findings according to gender (51.7% girls). Weighted means were used in the review.

**Zhang et al. (2018)**

For physical activity, the study reported an OR of 0.48 for regular activity compared to low activity, which was inverted to an OR of 2.08 for low activity.

## Zhang et al. (2022)

The study reported separate figures for TV viewing on weekdays and weekends. The review used mean values.

# Appendix B – Regression models for study quality variables

| **Table A1: Linear regression models for study quality variables (across behaviors and disorders).** | | | | | | |
| --- | --- | --- | --- | --- | --- | --- |
|  | **Full dataset** | | **All cross-sectional studies** | | **All longitudinal (behavior before disorder) studies** | |
|  | Beta | Sig. | Beta | Sig. | Beta | Sig. |
| Sample size | **-.078** | **.063** | **-.088** | **.061** | **-.393** | **.010** |
| Specialty sample | .016 | .705 | .003 | .948 | -.130 | .388 |
| Problem behavior | **.123** | **.004** | **.148** | **.002** | -.023 | .856 |
| Misery adj. | -.051 | .224 | -.034 | .479 | -.079 | .536 |
| Trauma adj. | -.068 | .105 | **-.082** | **.077** | -.076 | .565 |
| R^2^ | 0.027 | | 0.034 | | 0.124 | |
| Note: Associations at marginal significance (p < .1) or better marked in bold. | | | | | | |

| **Table A2: Linear regression models for study quality variables (cross-sectional studies stratified by behavior).** | | | | | | | | |
| --- | --- | --- | --- | --- | --- | --- | --- | --- |
|  | **Cannabis use** | | **Tobacco use** | | **Sedentary behavior** | | **Poor diet quality** | |
|  | Beta | Sig. | Beta | Sig. | Beta | Sig. | Beta | Sig. |
| Sample size | -.113 | .190 | -.068 | .385 | -.091 | .331 | -.167 | .214 |
| Specialty sample | **-.212** | **.014** | .073 | .380 | -.048 | .638 | .073 | .595 |
| Problem behavior | .093 | .281 | **.133** | **.098** | **.225** | **.034** | **.456** | **.004** |
| Misery adj. | -.106 | .218 | **.159** | **.054** | -.023 | .845 | -.084 | .545 |
| Trauma adj. | -.119 | .170 | -.033 | .691 | -.107 | .307 | -.084 | .511 |
| Cannabis adj. | --- | --- | **-.203** | **.013** | -.075 | .453 | **-.295** | **.069** |
| Tobacco adj. | **-.284** | **.001** | **---** | **---** | -.155 | .146 | -.212 | .168 |
| Sed./diet adj. | .014 | .870 | -.077 | .339 | --- | --- | --- | --- |
| R^2^ | 0.131 | | 0.090 | | 0.141 | | 0.275 | |
| Note: Associations at marginal significance (p < .1) or better marked in bold. | | | | | | | | |

| **Table A3a: Linear regression models for study quality variables (cross-sectional studies stratified by disorder).** | | | | | | | | |
| --- | --- | --- | --- | --- | --- | --- | --- | --- |
|  | **Psychosis** | | **Depression** | | **Anxiety** | | **Bipolar disorder** | |
|  | Beta | Sig. | Beta | Sig. | Beta | Sig. | Beta | Sig. |
| Sample size | -.025 | .875 | -.051 | .461 | -.039 | .731 | **-.454** | **.056** |
| Specialty sample | -.041 | .823 | .043 | .541 | .078 | .476 | -.251 | .287 |
| Problem behavior | **.279** | **.097** | **.167** | **.017** | -.020 | .860 | **.541** | **.015** |
| Misery adj. | -.073 | .726 | -.025 | .726 | -.129 | .241 | -.104 | .613 |
| Trauma adj. | -.263 | .145 | -.057 | .415 | -.077 | .477 | .034 | .867 |
| R^2^ | 0.180 | | 0.035 | | 0.029 | | 0.375 | |
| Note: Associations at marginal significance (p < .1) or better marked in bold. | | | | | | | | |

| **Table A3b: Linear regression models for study quality variables (cross-sectional studies stratified by disorder).** | | | | | | |
| --- | --- | --- | --- | --- | --- | --- |
|  | **Personality disorder** | | **ADHD** | | **PTSD** | |
|  | Beta | Sig. | Beta | Sig. | Beta | Sig. |
| Sample size | -.114 | .952 | -.044 | .801 | -.072 | .721 |
| Specialty sample | .004 | .998 | -.240 | .202 | -.048 | .800 |
| Problem behavior | .012 | .980 | **.397** | **.043** | .035 | .863 |
| Misery adj. | --- | --- | .160 | .396 | -.101 | .602 |
| Trauma adj. | --- | --- | **-.364** | **.065** | -.110 | .561 |
| R^2^ | 0.014 | | 0.201 | | 0.037 | |
| Note: Associations at marginal significance (p < .1) or better marked in bold. | | | | | | |

# Appendix C – Supplementary figures

Figure A2a. Odds ratios for longitudinal studies of cannabis and psychiatric disorders:


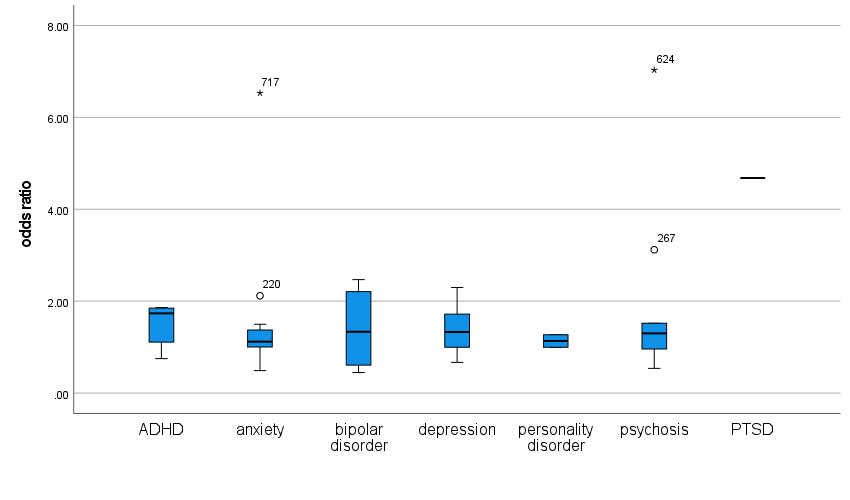


Figure A2b. Odds ratios for longitudinal (behavior before disorder) studies of cannabis and psychiatric disorders:


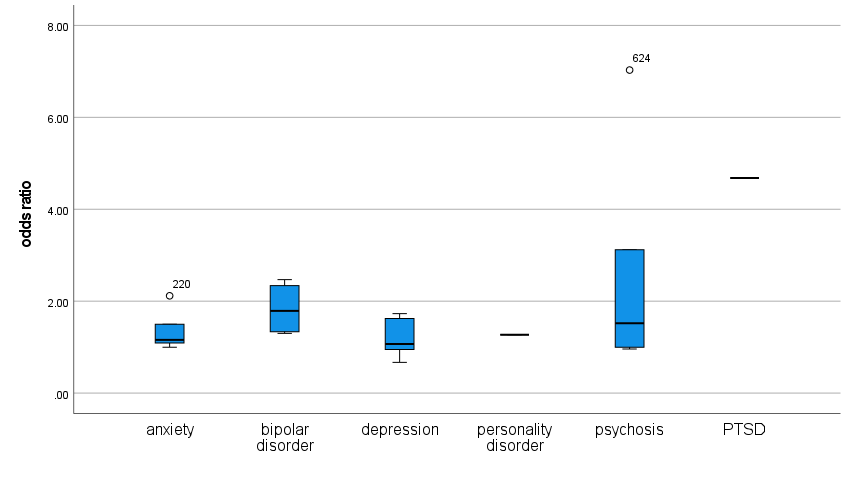


Figure A2c. Odds ratios for cross-sectional studies of tobacco and psychiatric disorders:


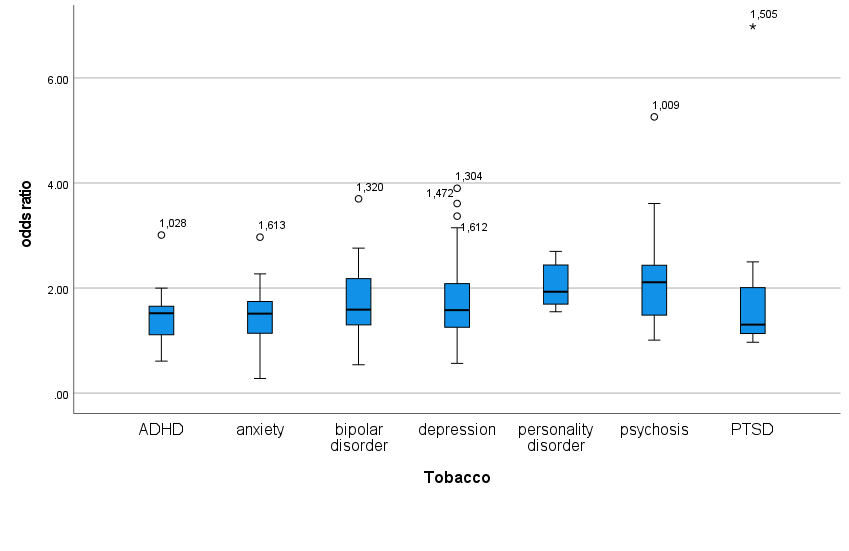


Figure A2d. Odds ratios for cross-sectional studies of sedentary behavior/diet quality and psychiatric disorders:


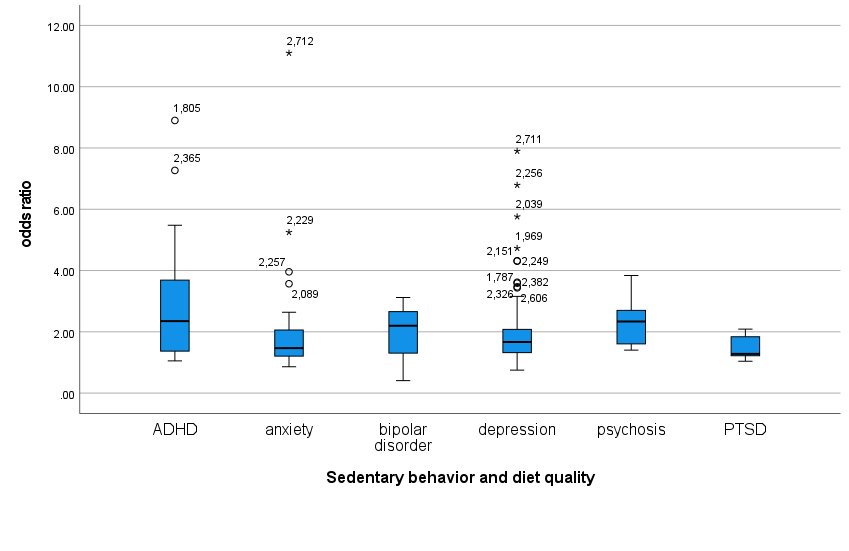


Note: In order to improve the quality of presentation, one outlier study (Sze et al., 2021) with a very high OR of 28.19 for depression has been removed.

Figure A3. Odds ratios for psychosis in longitudinal (behavior before disorder) studies:


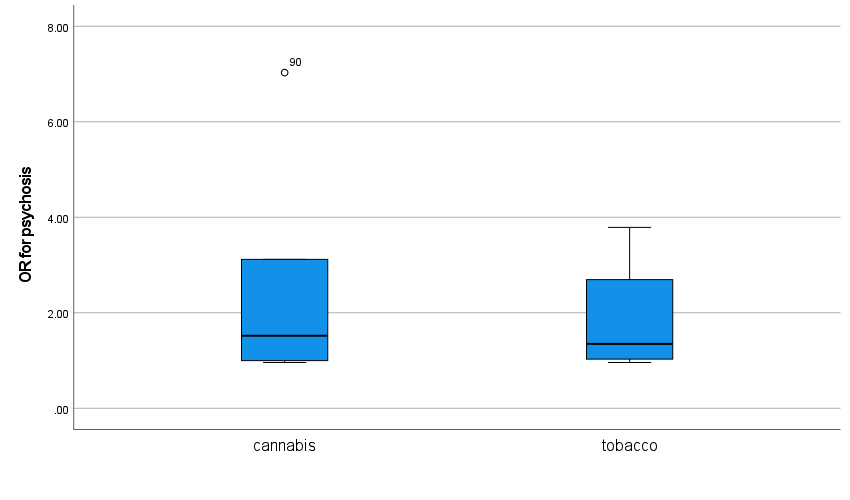


Figure A4. Odds ratios for depression in longitudinal (behavior before disorder) studies:


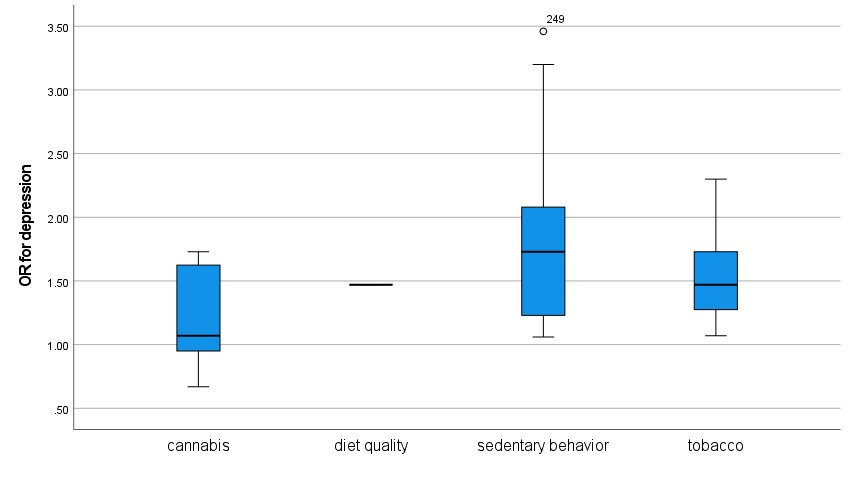


Figure A5. Odds ratios for anxiety in longitudinal (behavior before disorder) studies:


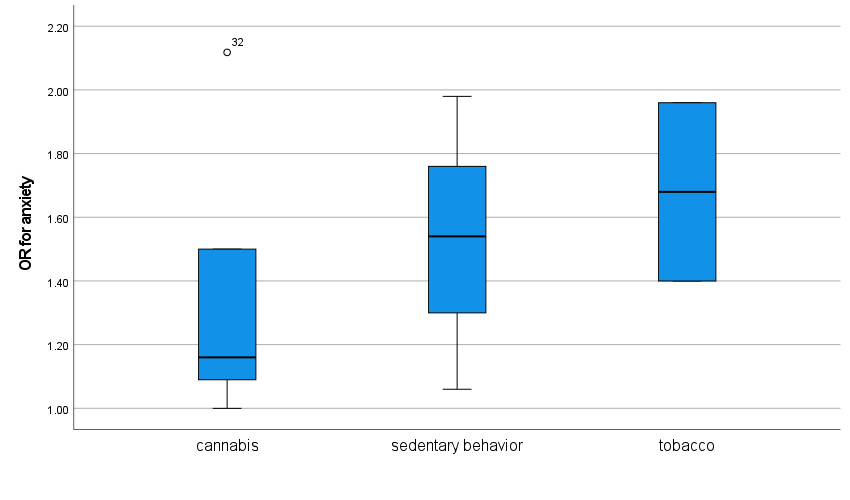


Figure A6. Odds ratios for bipolar disorder in longitudinal (behavior before disorder) studies:


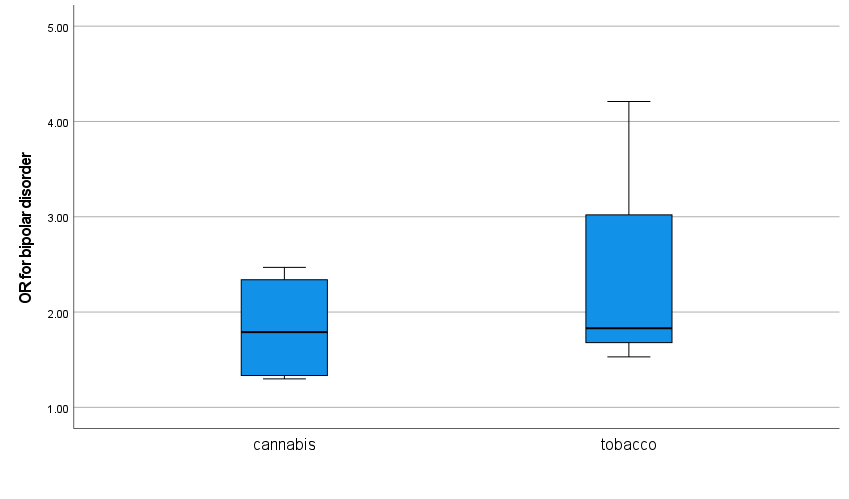


# Appendix D – Supplementary tables

| **Table A9: Associations between cannabis and psychiatric disorders compared to tobacco** | | | | |
| --- | --- | --- | --- | --- |
|  |  |  |  |  |
|  | **Personality disorder** | **ADHD** | **Bipolar disorder** | **PTSD** |
| **Cannabis stronger**  **(OR + 0.25)** | Cougle et al. (2016: CS)  Estévez et al. (2016)  Holzer et al. (2022) | Capusan et al. (2019)  Welsh et al. (2017) | Patel et al. (2022) | Ehlers et al. (2016) |
| **Cannabis and tobacco on same level** |  | Estévez et al. (2016)  Moulin et al. (2018) |  |  |
| **Tobacco stronger**  **(OR + 0.25)** |  |  | Cougle et al. (2016: CS) | Welsh et al. (2017) |
|  |  |  |  |  |
| Abbreviations: CS cross-sectional; LN longitudinal | | | | |

| **Table A10: Associations between cannabis/tobacco and psychiatric disorders compared to diet quality and sedentary behavior** | | | | |
| --- | --- | --- | --- | --- |
|  |  |  |  |  |
|  | **ADHD** | **PTSD** |  |  |
| **Cannabis/tobacco stronger (OR + 0.25)** | Moulin et al. (2018) |  |  |  |
| **Cannabis/tobacco and diet quality/sedentary behavior on same level** |  |  |  |  |
| **Diet quality/sedentary behavior stronger**  **(OR + 0.25)** | Weissenberger et al. (2018) | Hruby et al. (2021)  Whitworth et al. (2022) |  |  |
|  |  |  |  |  |
|  | | | | |
